# Supplementary figures and images for: Functional hierarchy among different Rab27 effectors involved in secretory granule exocytosis
Source: eLife. 2023 Feb 21;12:e82821. doi: 10.7554/eLife.82821 (PMC9988257; doi:10.7554/eLife.82821)

# Source data 1

## Uncropped blot images of Figure 1B

(upper)

MW (kDa)

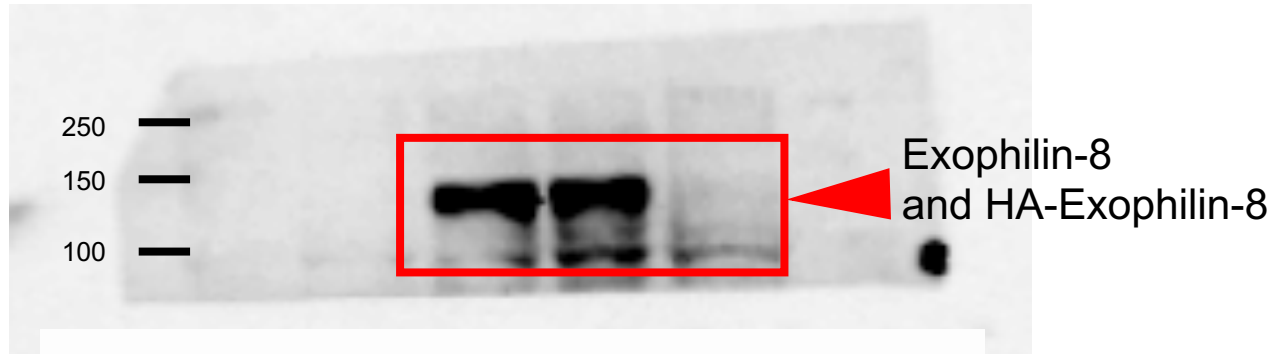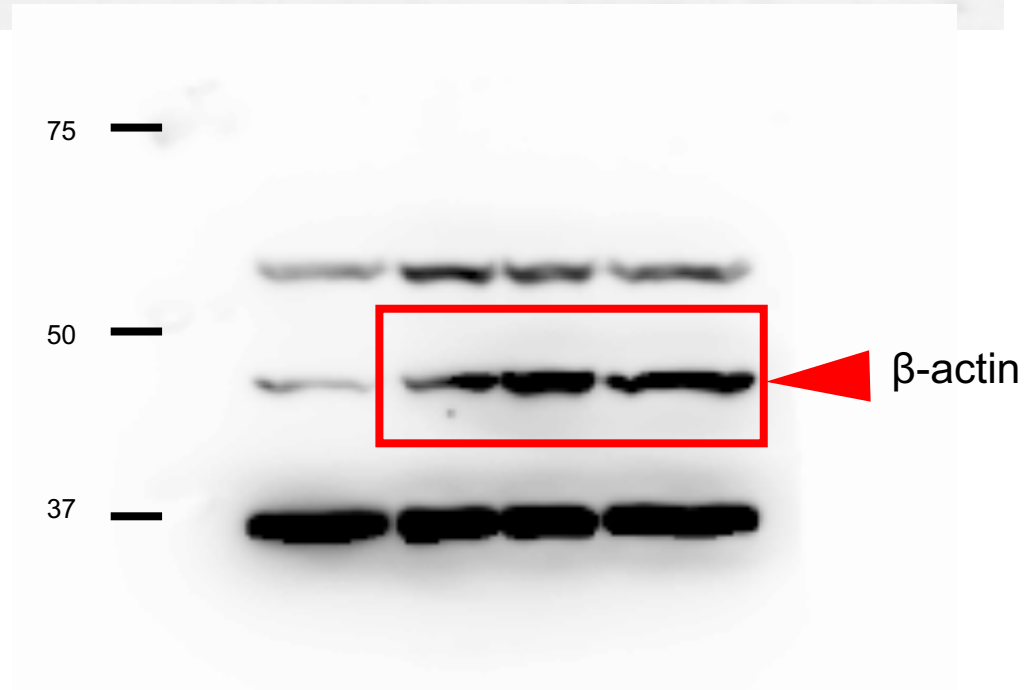

(lower)

MW (kDa)

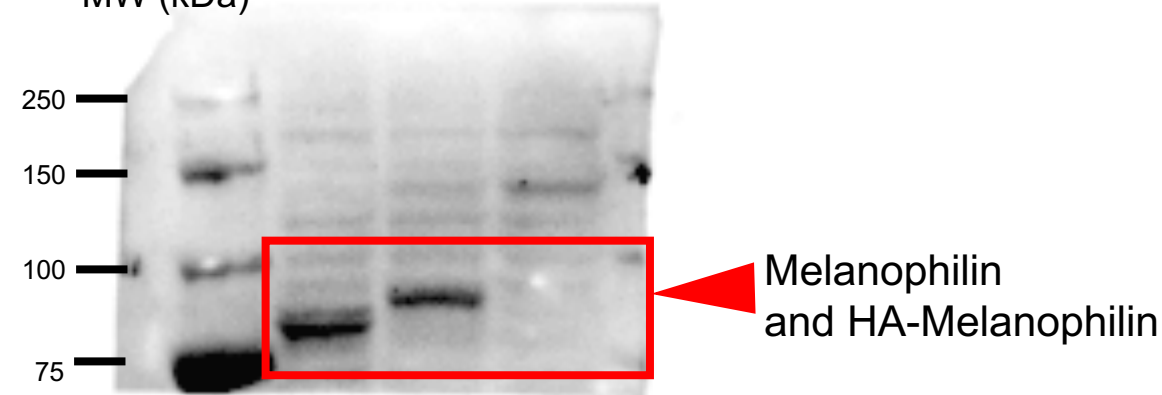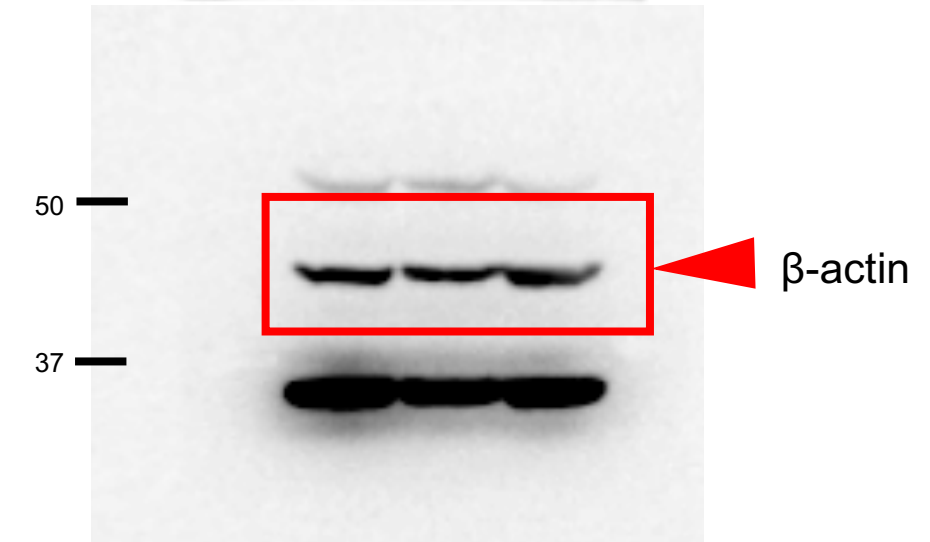

Supplement: Figure 1—source data 1. [file elife-82821-fig1-data1.zip › Figure 1-source data 1/Figure 1-Source Data 1.pdf]

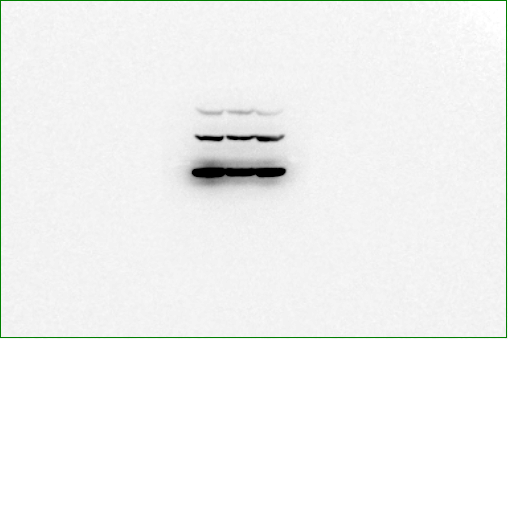

Supplement: Figure 1—source data 1. [file elife-82821-fig1-data1.zip › Figure 1-source data 1/Figure 1-Source Data 1 original files/Figure 1B ME8DKDKO rescued HA-MLPH actin.tif]

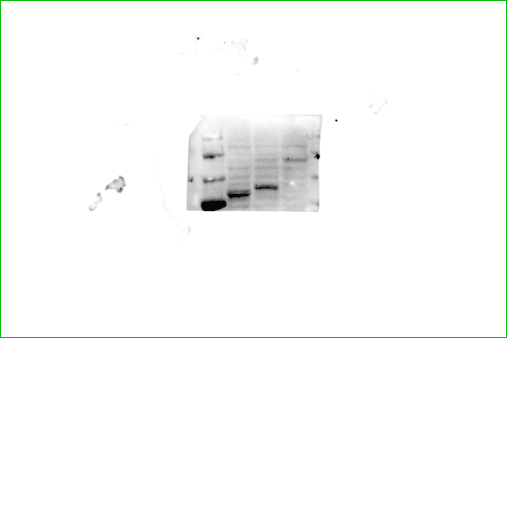

Supplement: Figure 1—source data 1. [file elife-82821-fig1-data1.zip › Figure 1-source data 1/Figure 1-Source Data 1 original files/Figure 1B ME8DKO rescued HA-MLPH.tif]

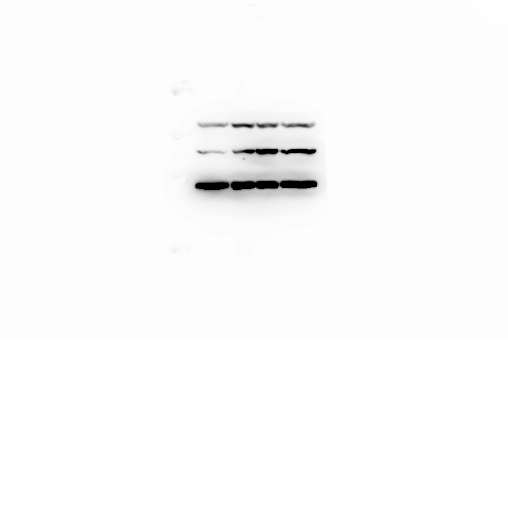

Supplement: Figure 1—source data 1. [file elife-82821-fig1-data1.zip › Figure 1-source data 1/Figure 1-Source Data 1 original files/Figure 1B ME8DKO rescued HA-Exo8 actin.tif]

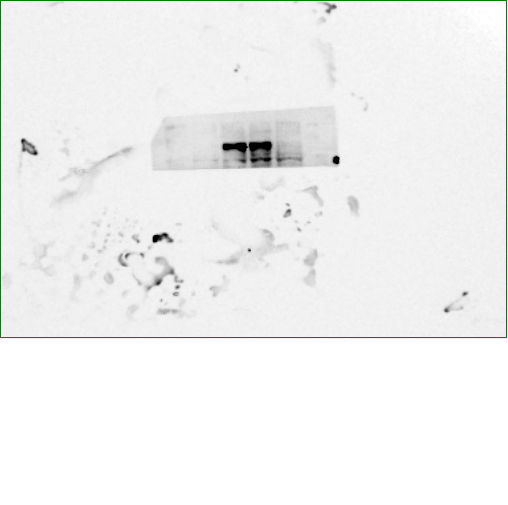

Supplement: Figure 1—source data 1. [file elife-82821-fig1-data1.zip › Figure 1-source data 1/Figure 1-Source Data 1 original files/Figure 1B ME8DKO rescued HA-Exo8.tif]

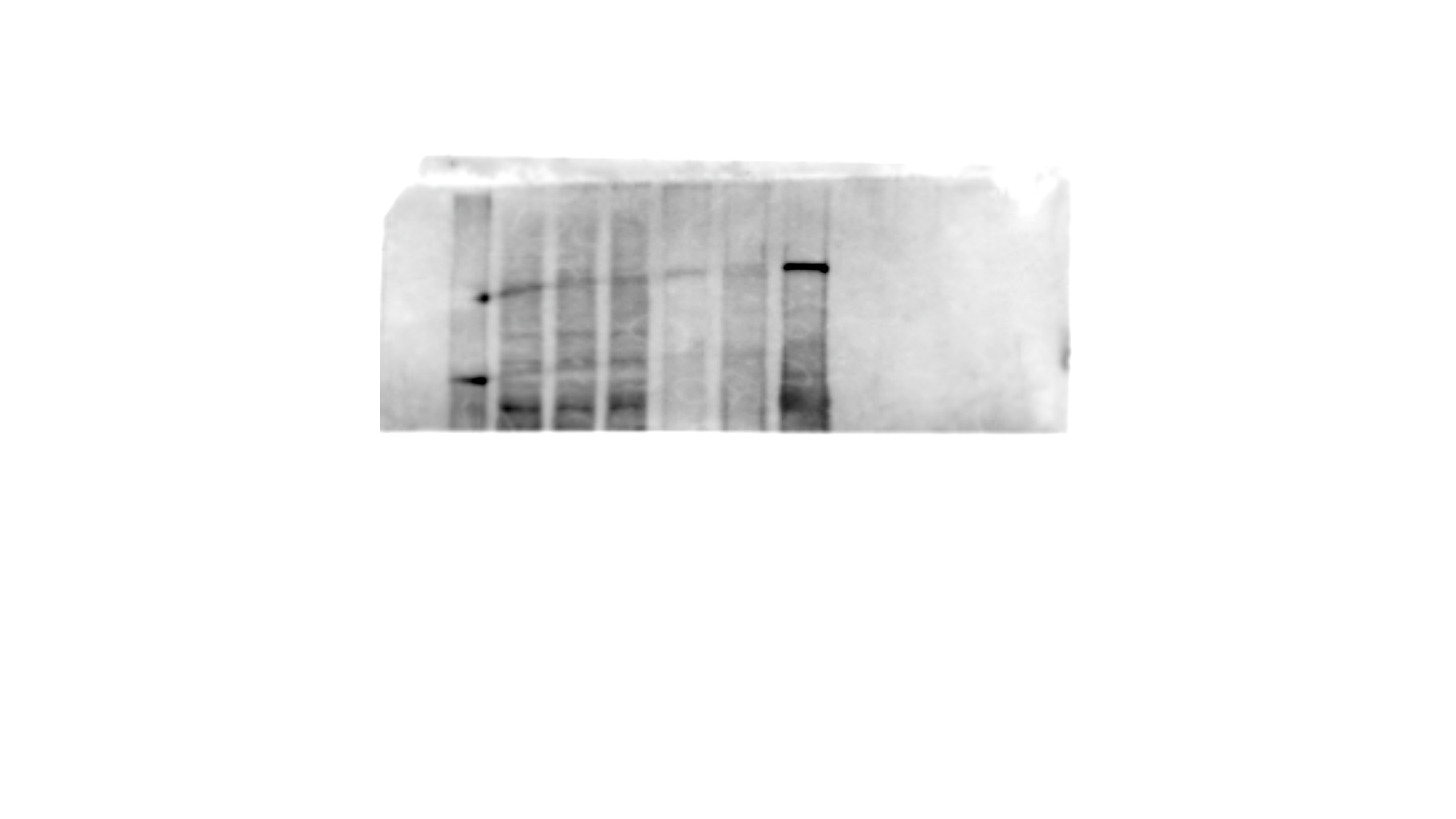

Supplement: Figure 3—source data 1. [file elife-82821-fig3-data1.zip › Figure 3-source data 2/Figure 3-source data 2 original files/Figure 3E Myosin7a.tif]

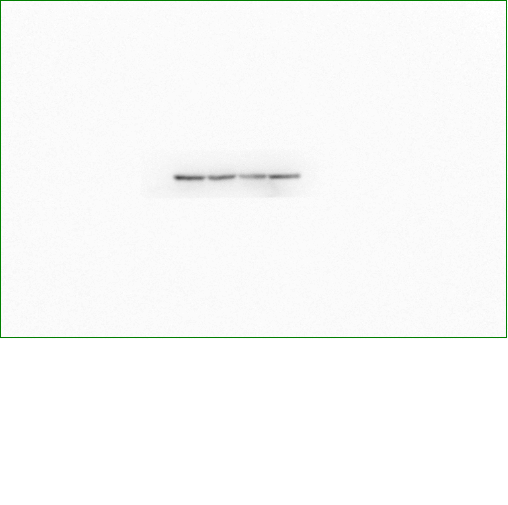

Supplement: Figure 3—source data 1. [file elife-82821-fig3-data1.zip › Figure 3-source data 2/Figure 3-source data 2 original files/Figure 3A Rab27a.tif]

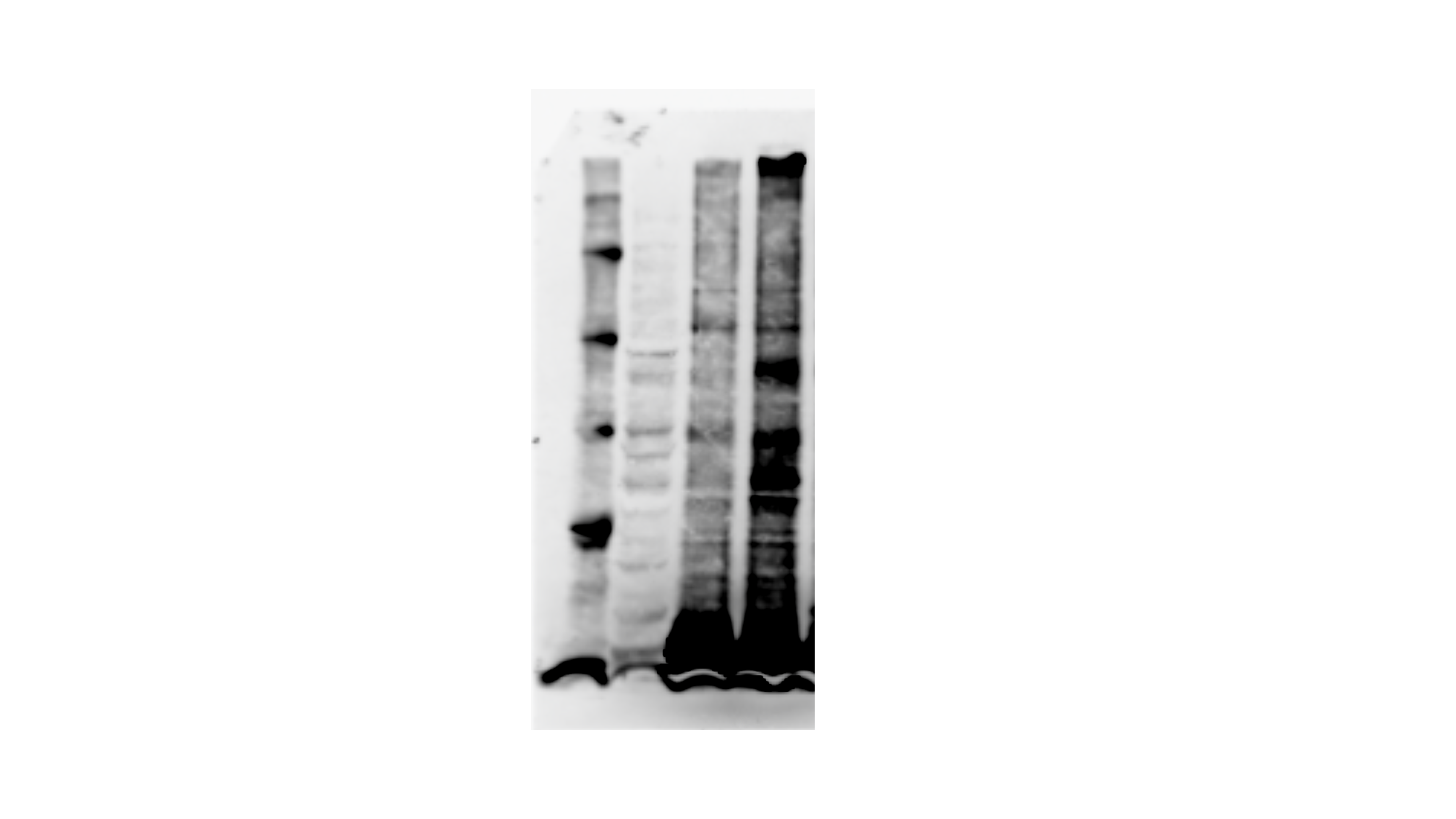

Supplement: Figure 3—source data 1. [file elife-82821-fig3-data1.zip › Figure 3-source data 2/Figure 3-source data 2 original files/Figure 3C Exo8.tif]

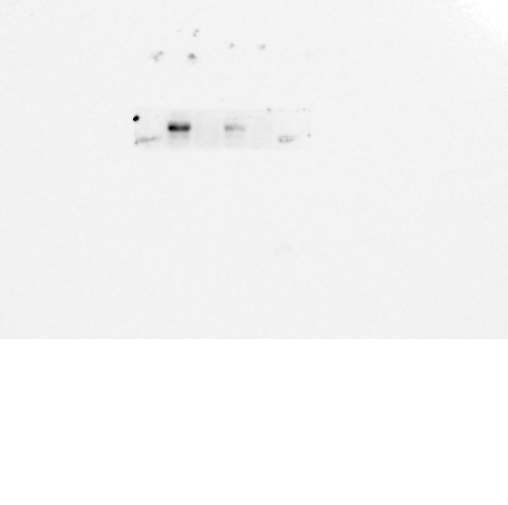

Supplement: Figure 3—source data 1. [file elife-82821-fig3-data1.zip › Figure 3-source data 2/Figure 3-source data 2 original files/Figure 3A MLPH.tif]

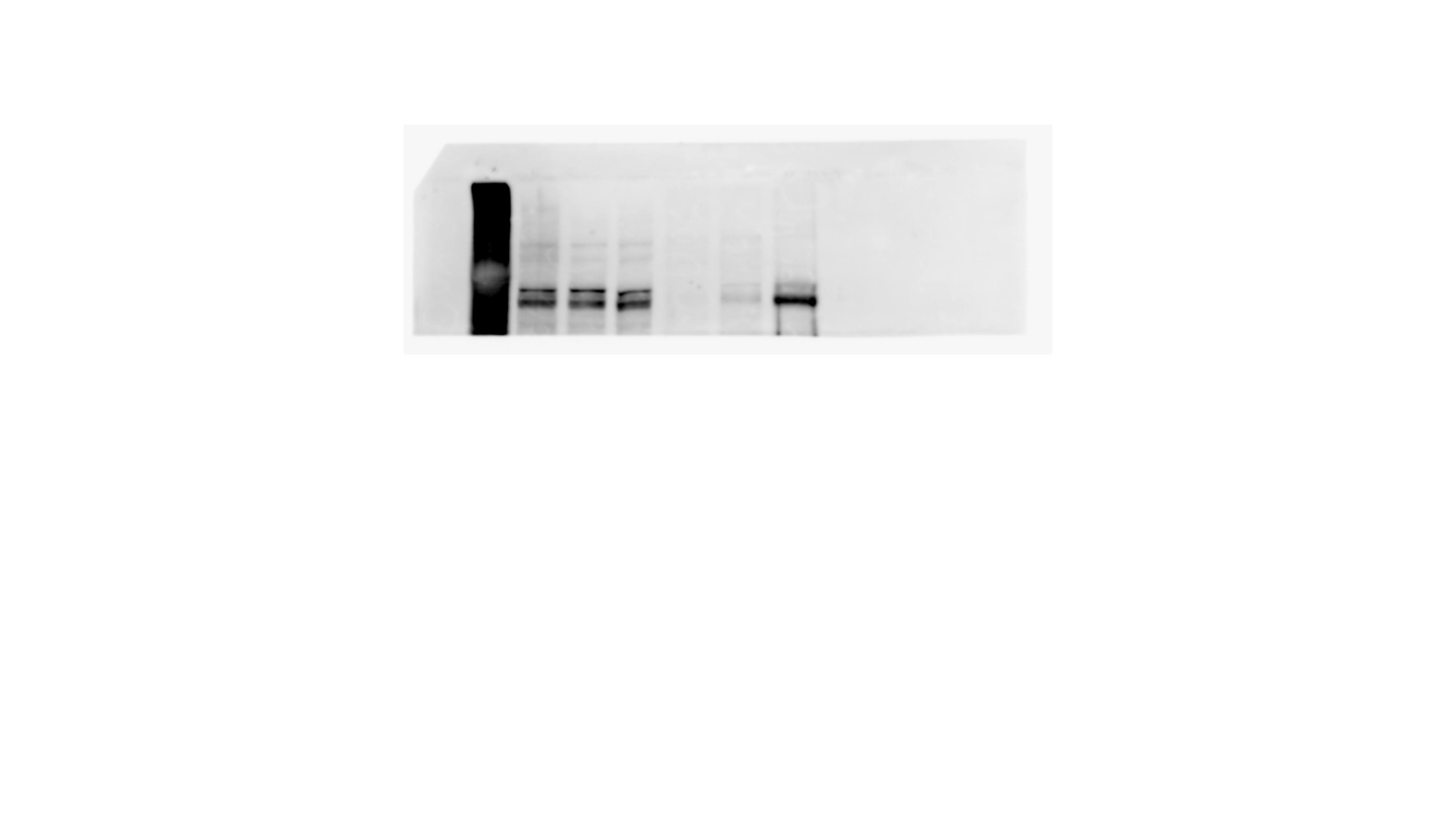

Supplement: Figure 3—source data 1. [file elife-82821-fig3-data1.zip › Figure 3-source data 2/Figure 3-source data 2 original files/Figure 3E RIM2.tif]

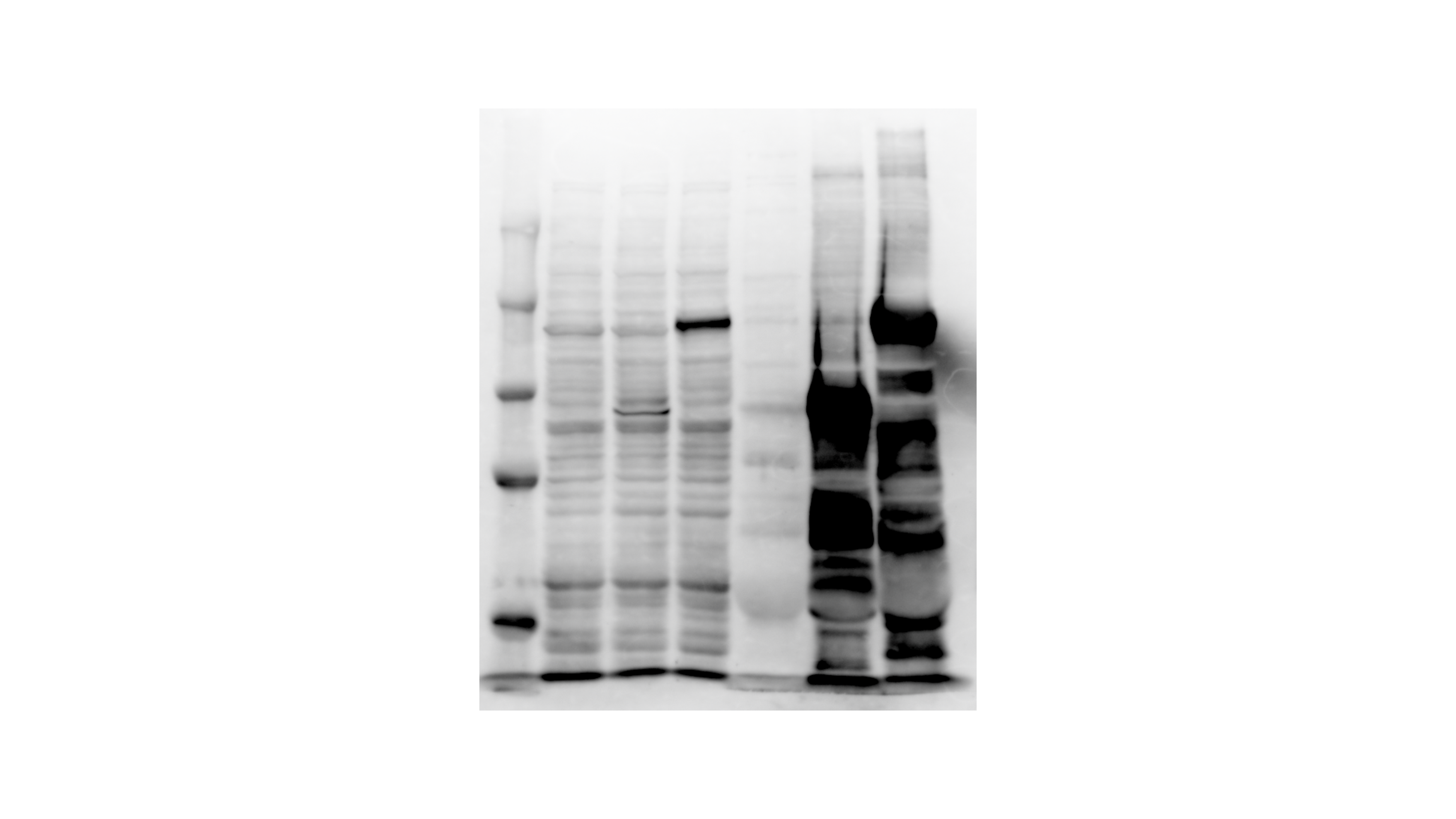

Supplement: Figure 3—source data 1. [file elife-82821-fig3-data1.zip › Figure 3-source data 2/Figure 3-source data 2 original files/Figure 3E Flag-Mlph and Flag-Exo8.tif]

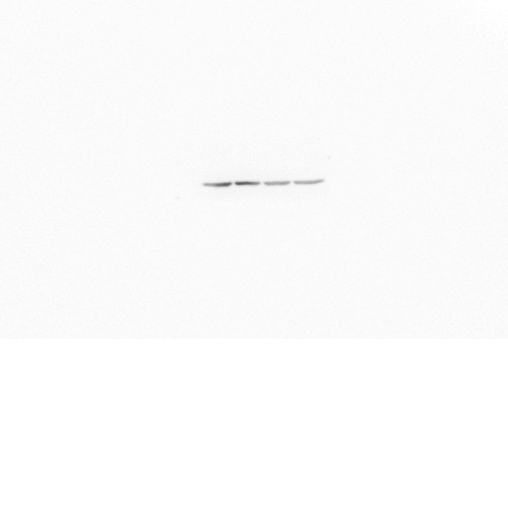

Supplement: Figure 3—source data 1. [file elife-82821-fig3-data1.zip › Figure 3-source data 2/Figure 3-source data 2 original files/Figure 3A actin.tif]

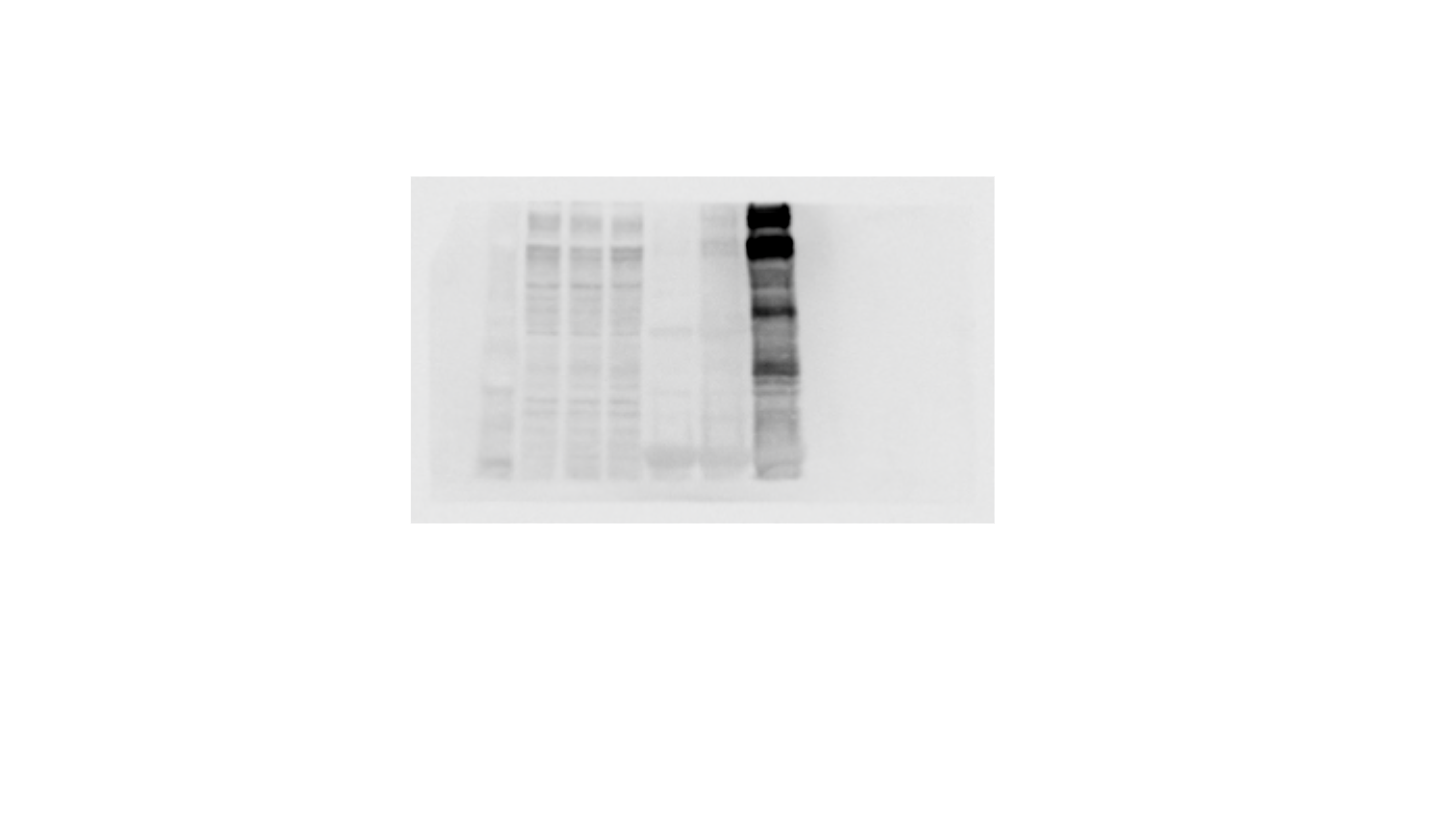

Supplement: Figure 3—source data 1. [file elife-82821-fig3-data1.zip › Figure 3-source data 2/Figure 3-source data 2 original files/Figure 3E RIMBP2.tif]

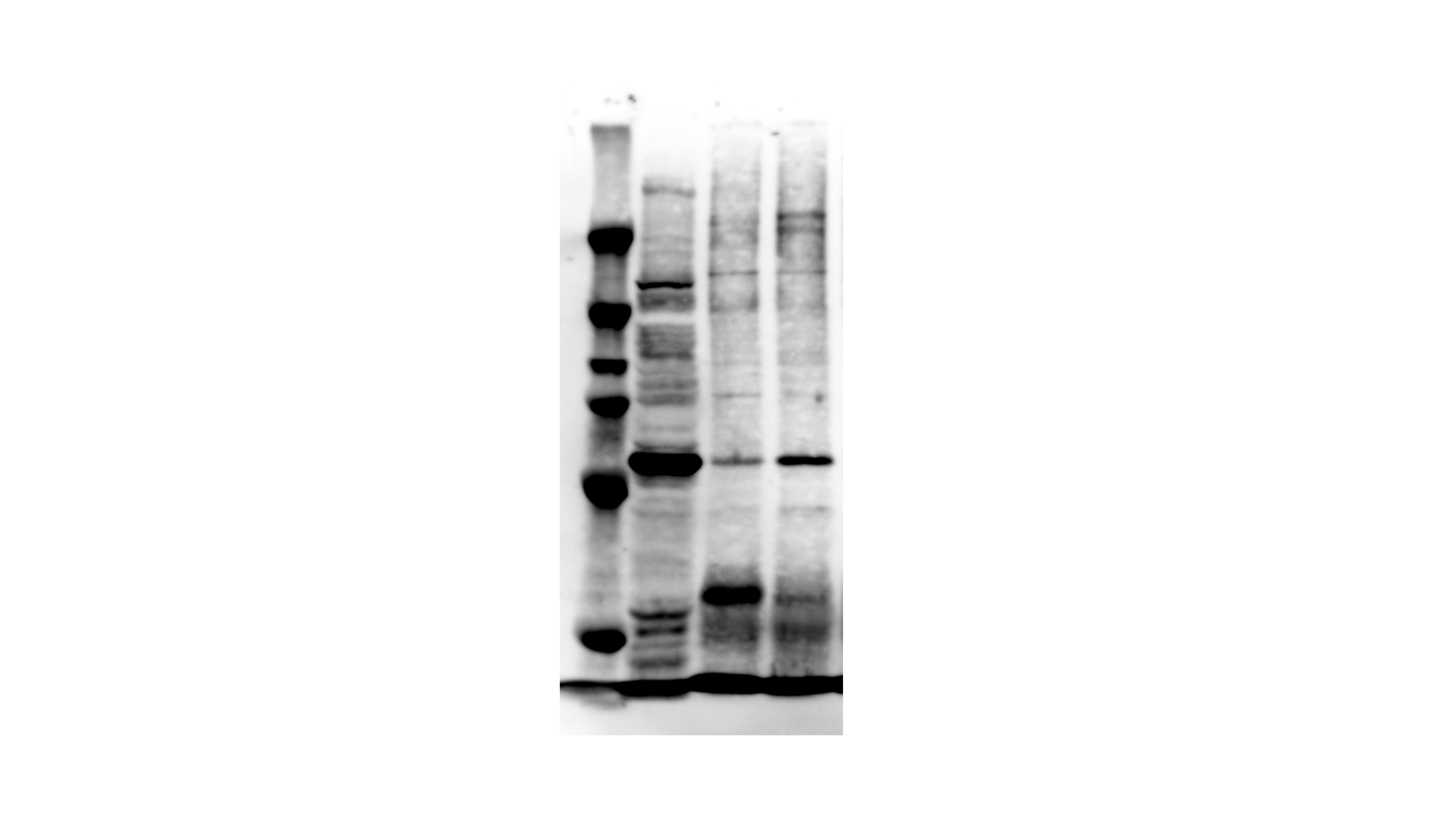

Supplement: Figure 3—source data 1. [file elife-82821-fig3-data1.zip › Figure 3-source data 2/Figure 3-source data 2 original files/Figure 3C SEC6.tif]

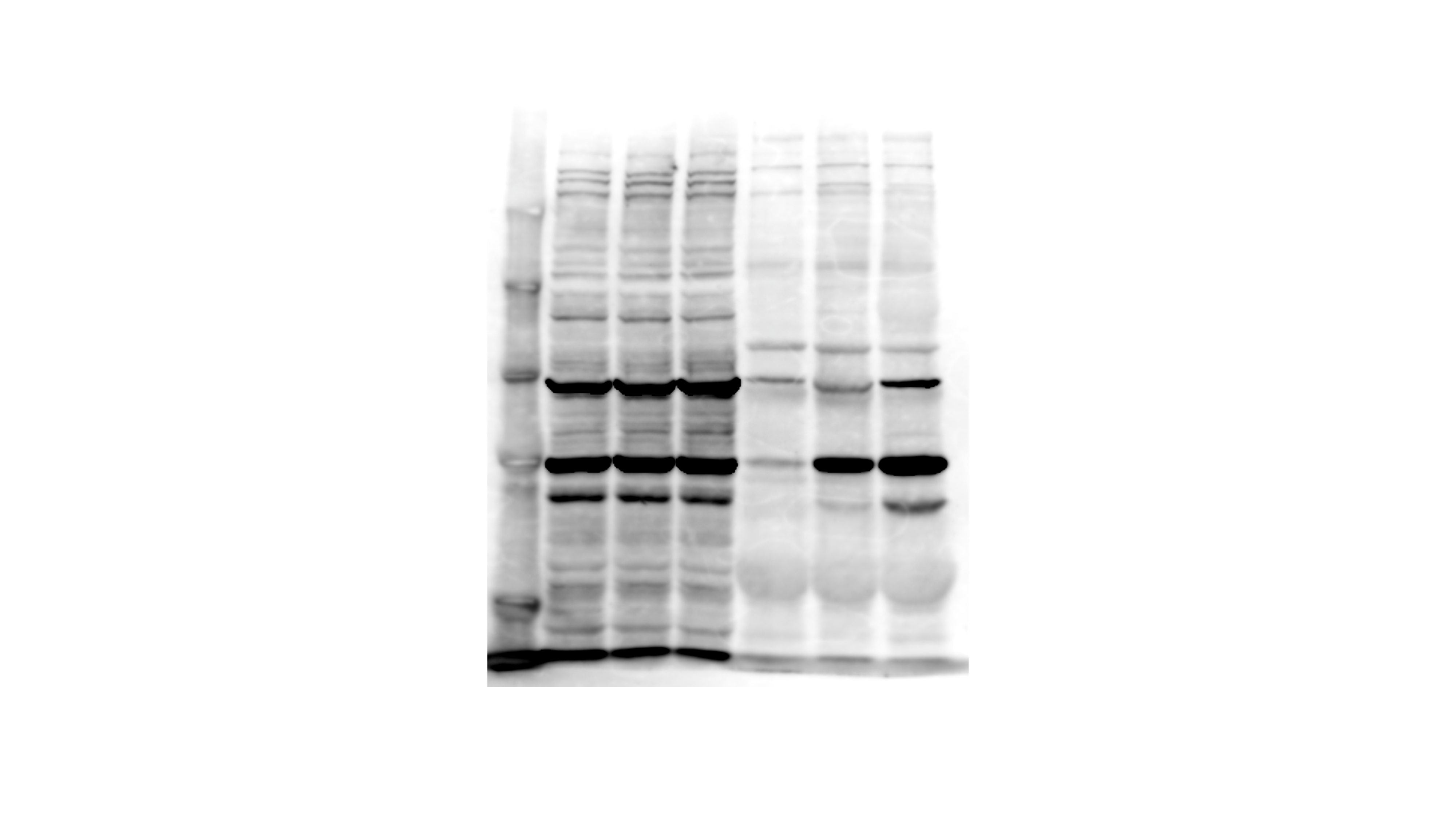

Supplement: Figure 3—source data 1. [file elife-82821-fig3-data1.zip › Figure 3-source data 2/Figure 3-source data 2 original files/Figure 3E SEC10.tif]

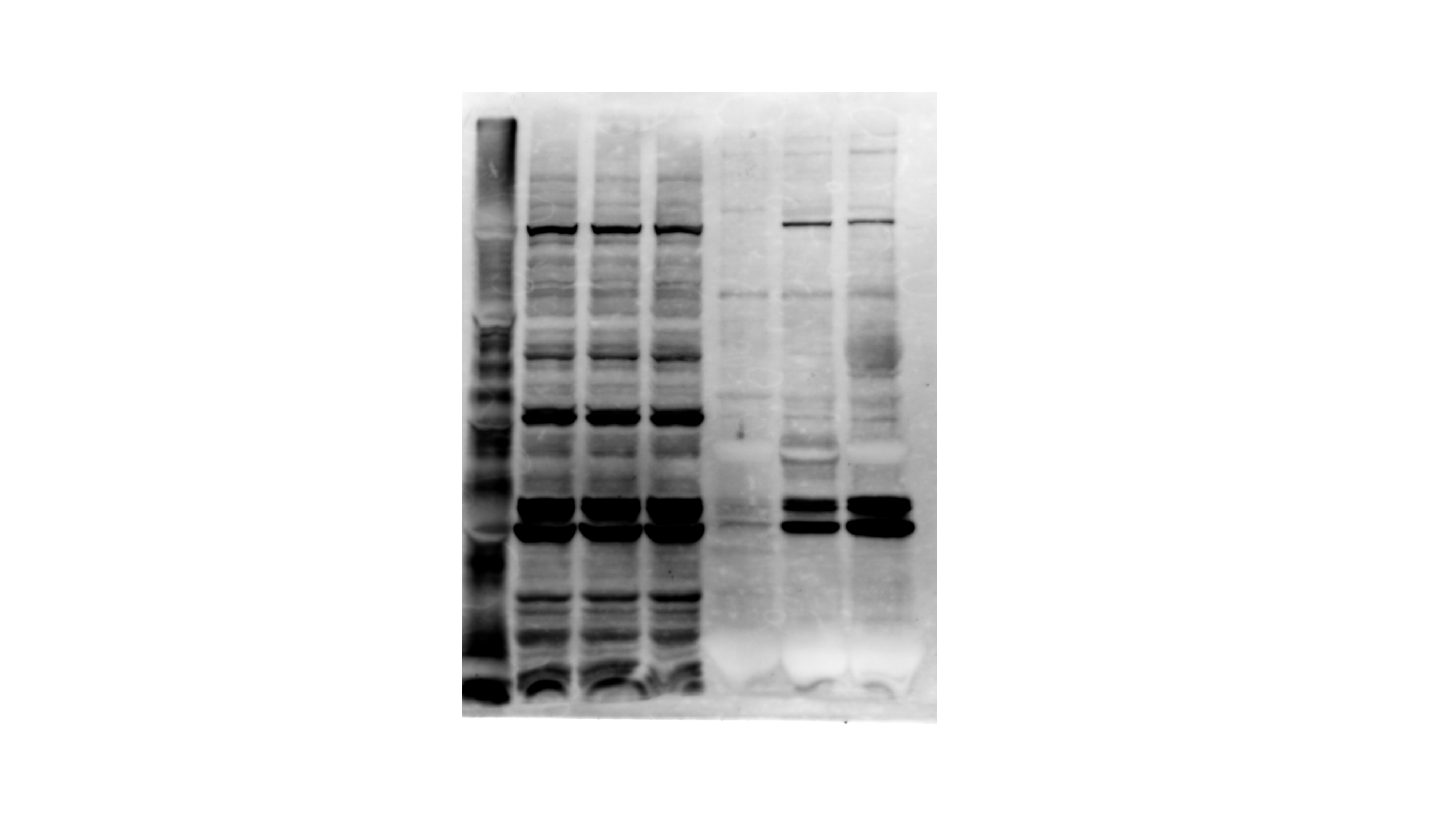

Supplement: Figure 3—source data 1. [file elife-82821-fig3-data1.zip › Figure 3-source data 2/Figure 3-source data 2 original files/Figure 3E EXO70.tif]

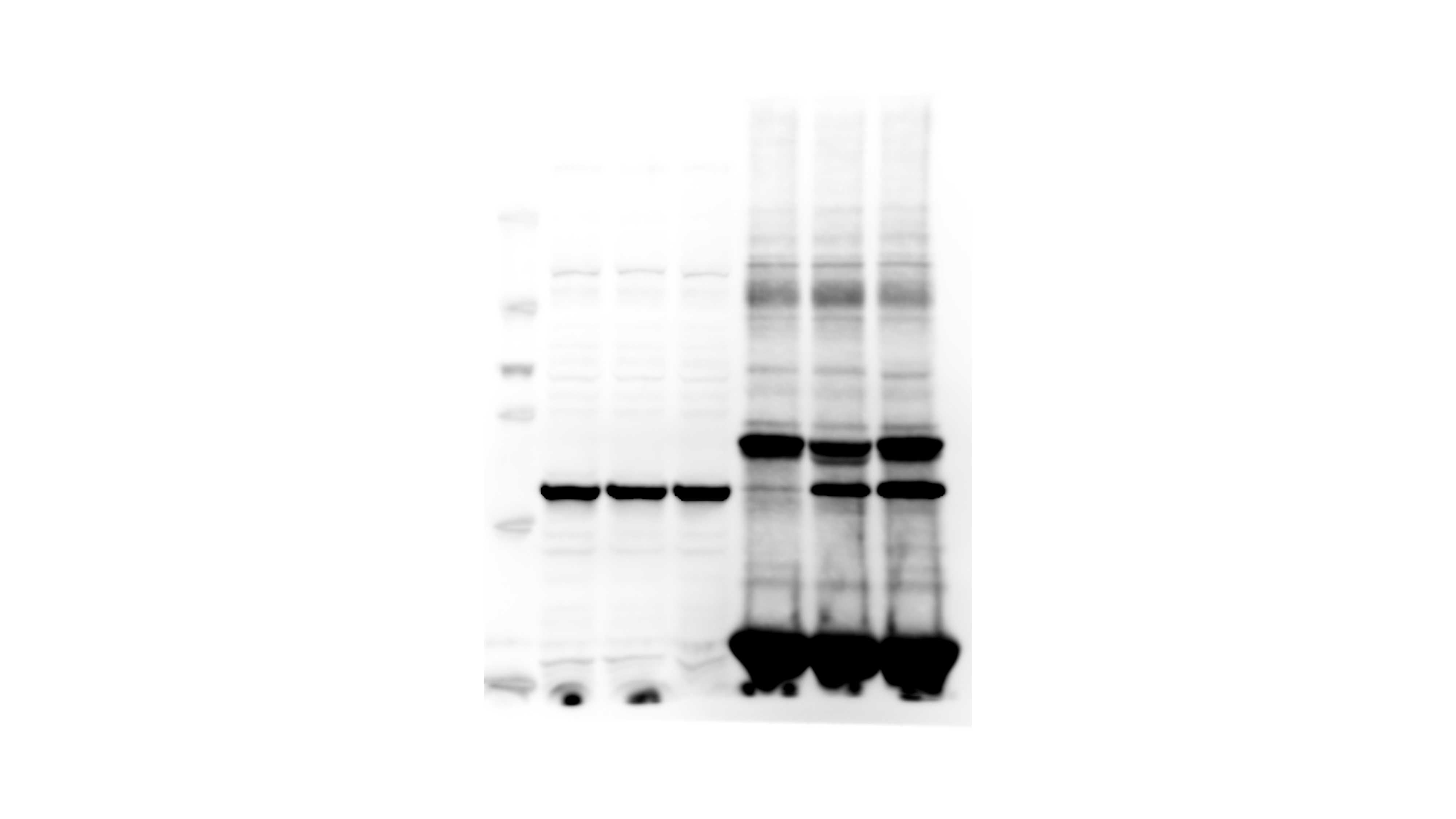

Supplement: Figure 3—source data 1. [file elife-82821-fig3-data1.zip › Figure 3-source data 2/Figure 3-source data 2 original files/Figure 3E SEC6.tif]

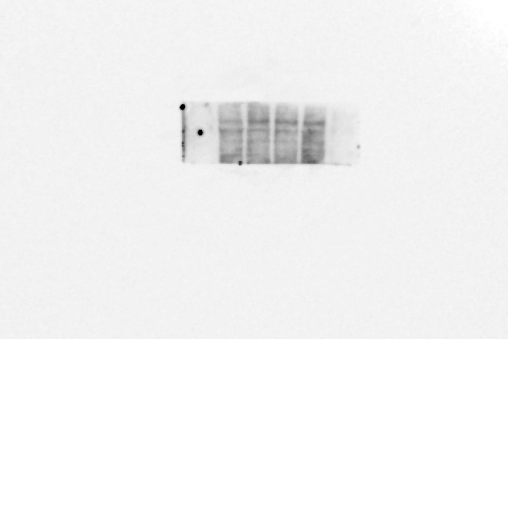

Supplement: Figure 3—source data 1. [file elife-82821-fig3-data1.zip › Figure 3-source data 2/Figure 3-source data 2 original files/Figure 3A Myosin7.tif]

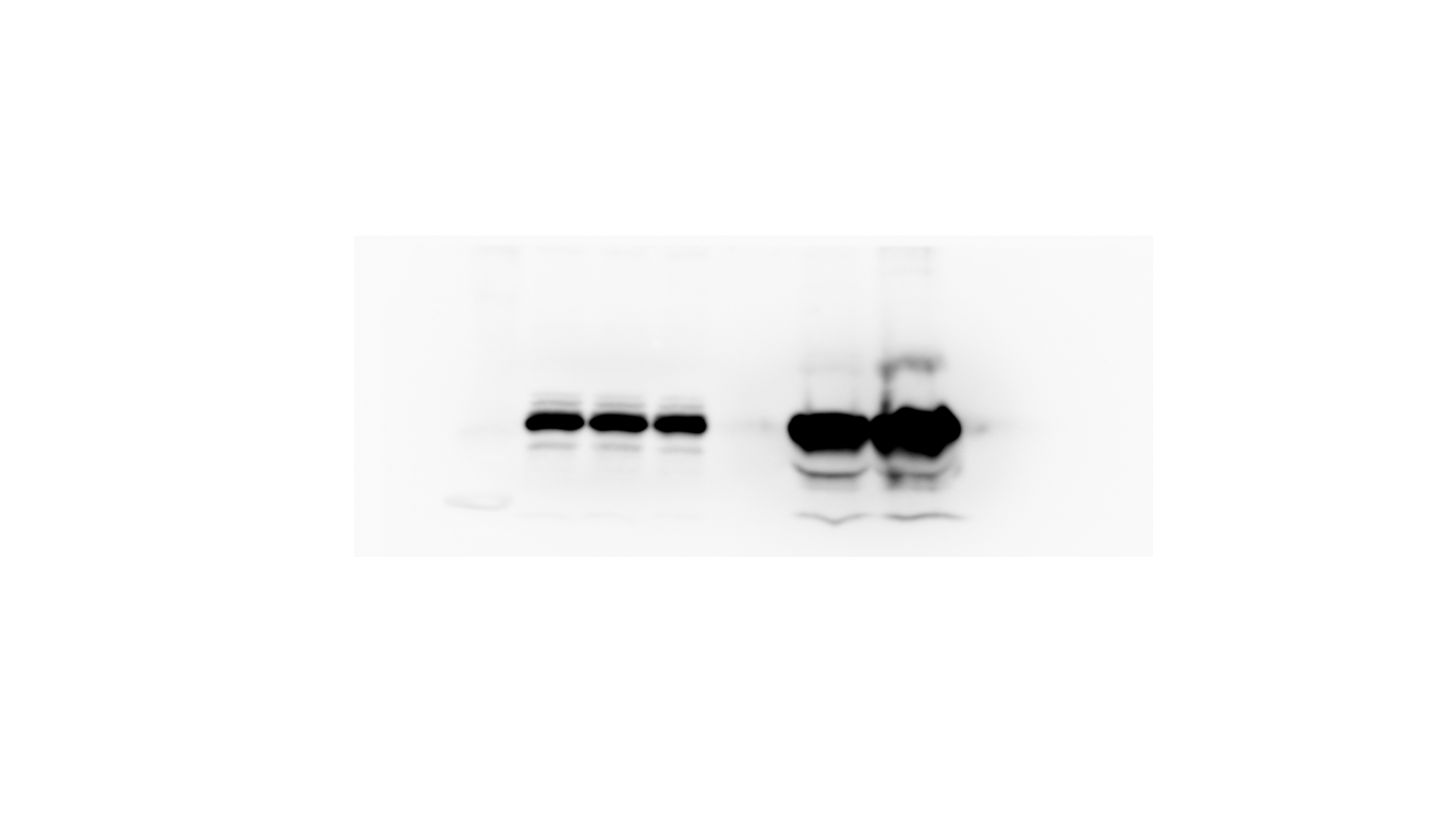

Supplement: Figure 3—source data 1. [file elife-82821-fig3-data1.zip › Figure 3-source data 2/Figure 3-source data 2 original files/Figure 3E Rab27a.tif]

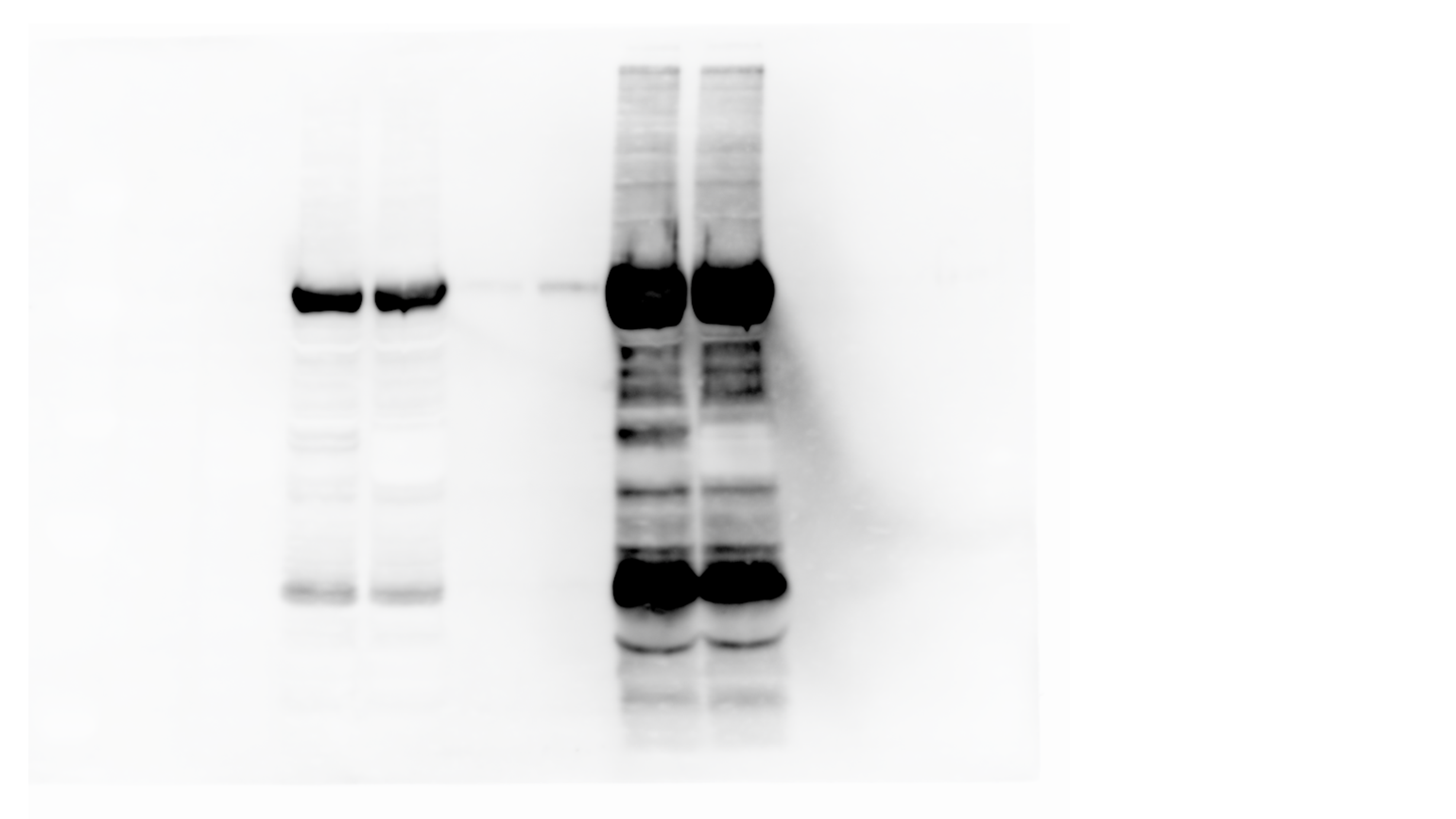

Supplement: Figure 3—source data 1. [file elife-82821-fig3-data1.zip › Figure 3-source data 2/Figure 3-source data 2 original files/Figure 3B mCherry-Exo8.tif]

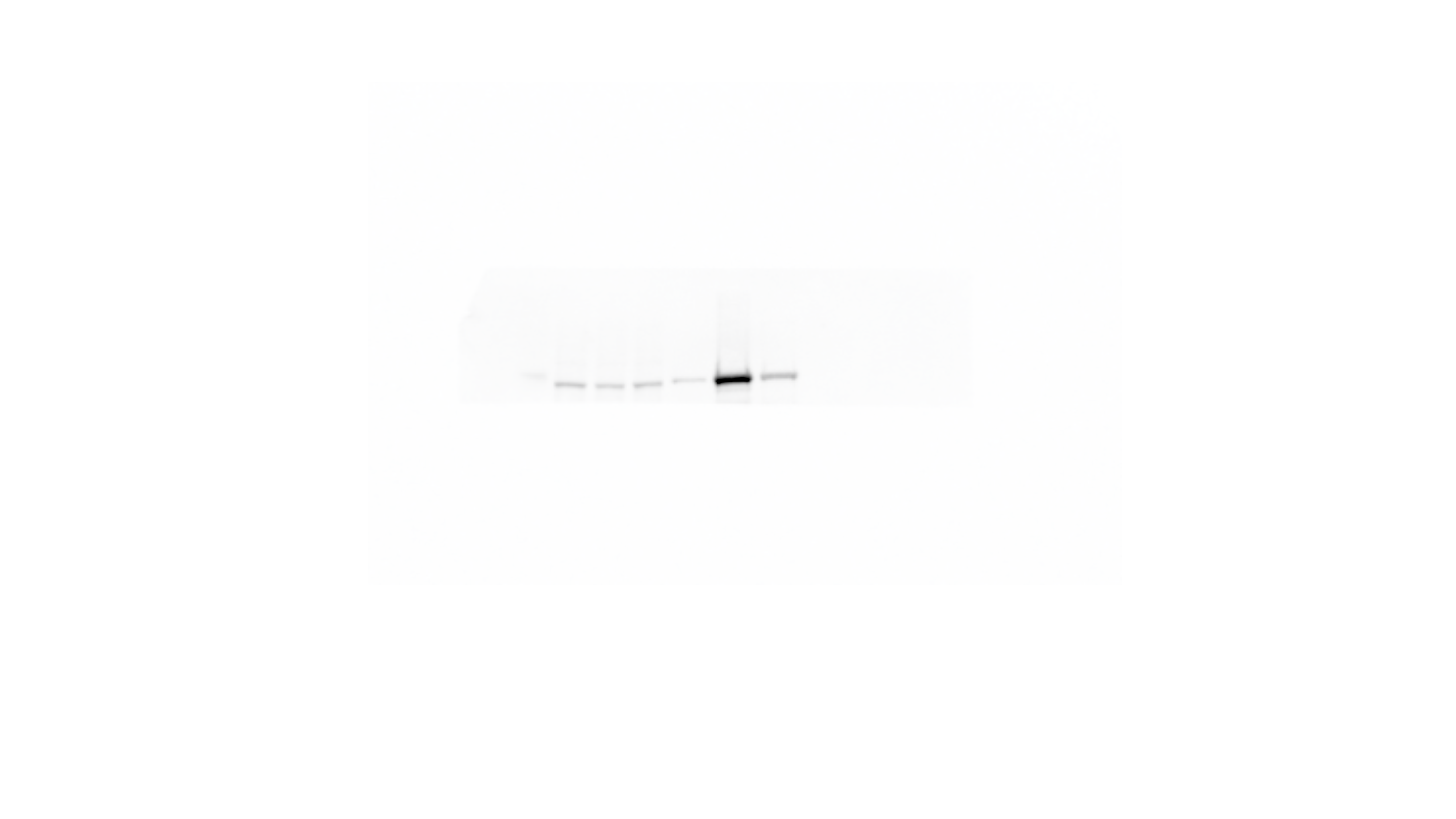

Supplement: Figure 3—source data 1. [file elife-82821-fig3-data1.zip › Figure 3-source data 2/Figure 3-source data 2 original files/Figure 3E Myosin5a.tif]

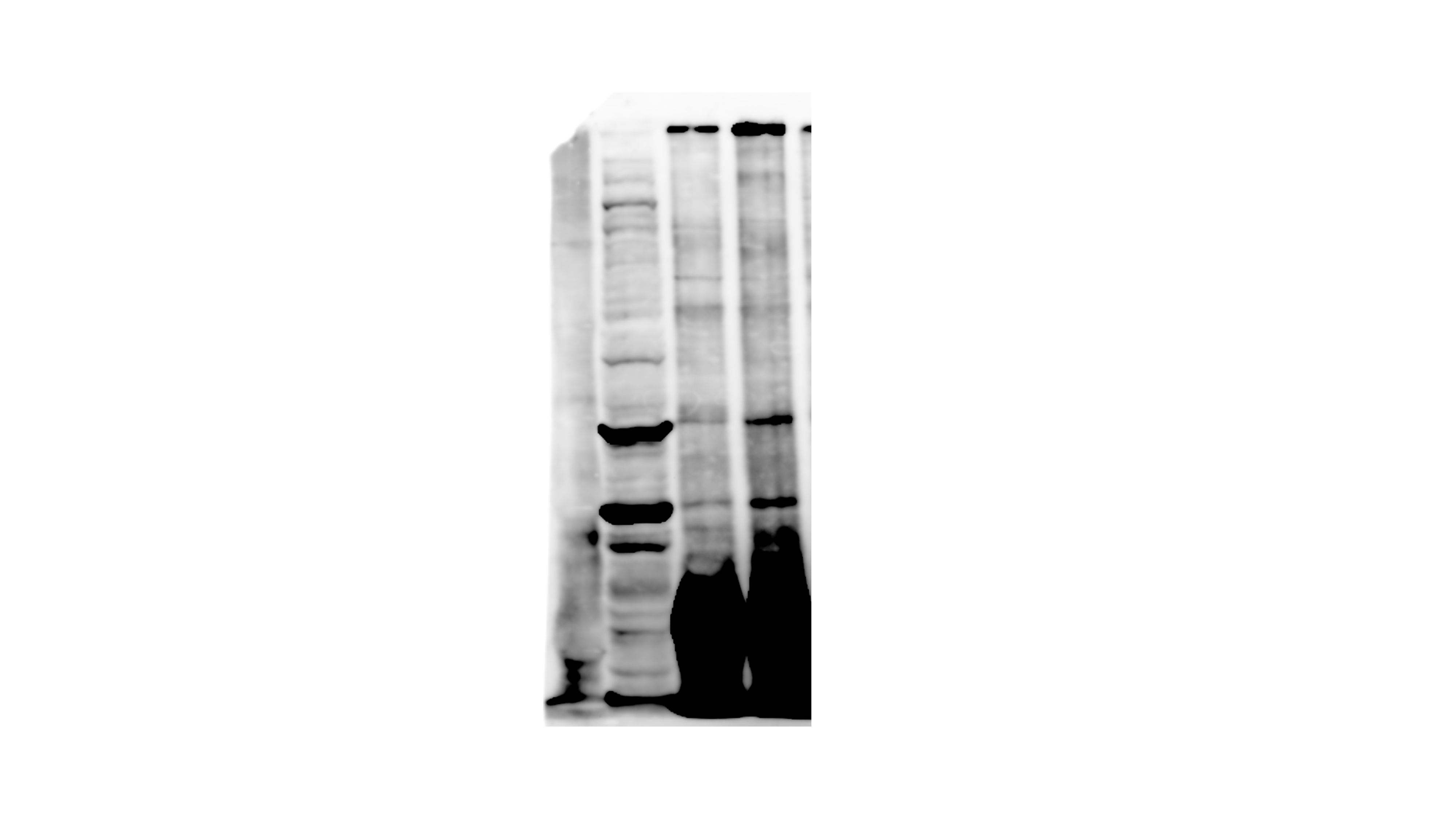

Supplement: Figure 3—source data 1. [file elife-82821-fig3-data1.zip › Figure 3-source data 2/Figure 3-source data 2 original files/Figure 3C SEC10.tif]

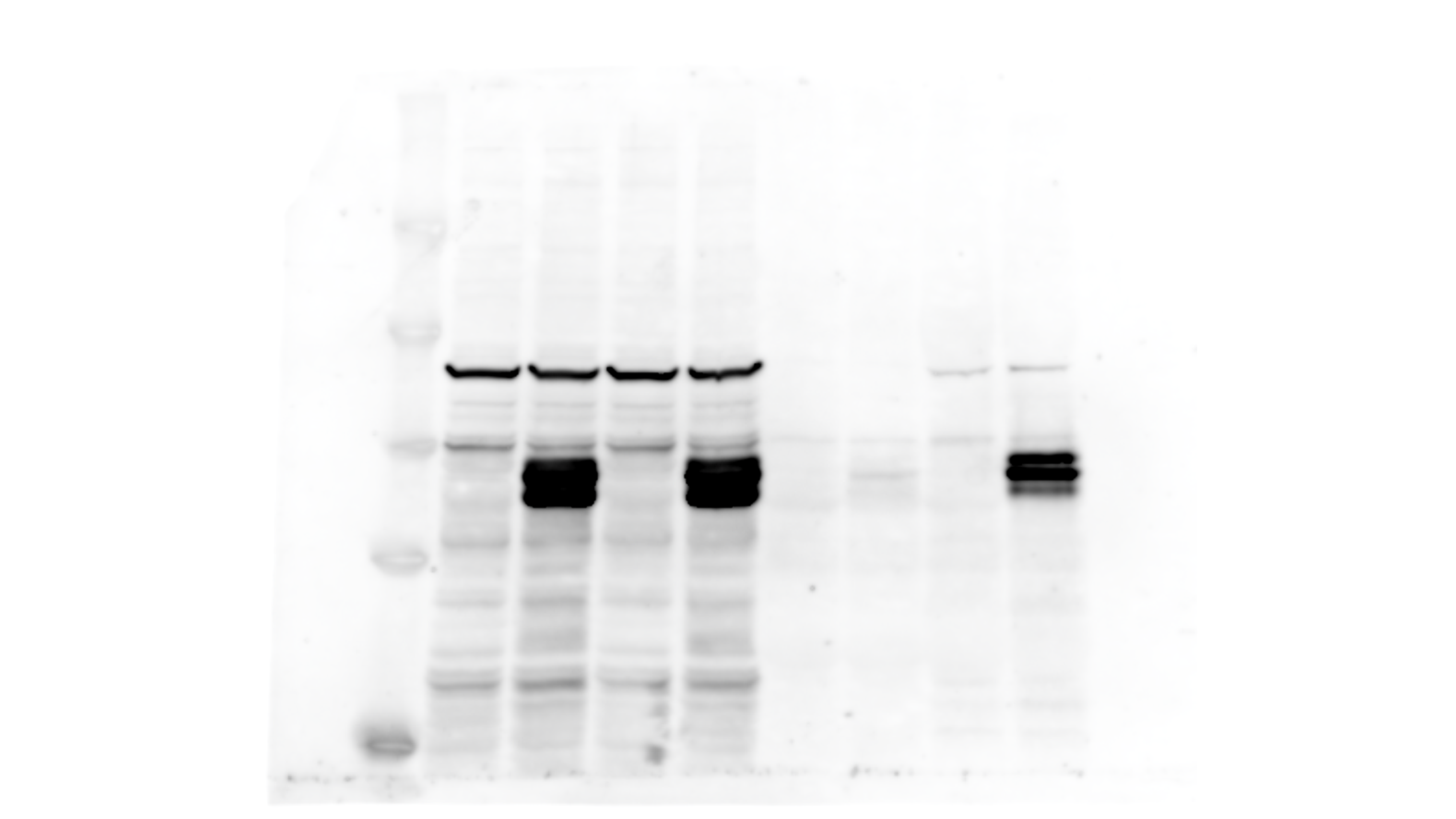

Supplement: Figure 3—source data 1. [file elife-82821-fig3-data1.zip › Figure 3-source data 2/Figure 3-source data 2 original files/Figure 3B Flag-Mlph.tif]

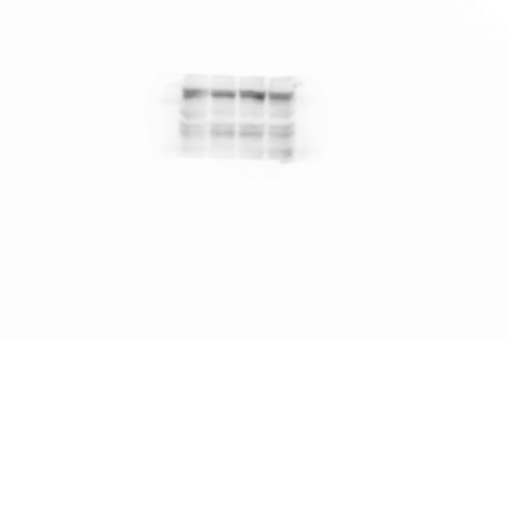

Supplement: Figure 3—source data 1. [file elife-82821-fig3-data1.zip › Figure 3-source data 2/Figure 3-source data 2 original files/Figure 3A Ganuphilin.tif]

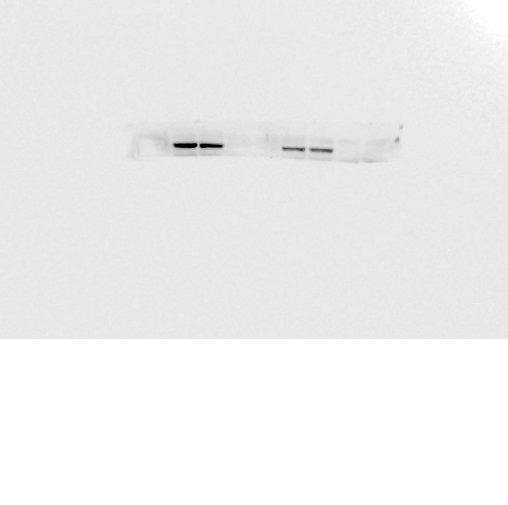

Supplement: Figure 3—source data 1. [file elife-82821-fig3-data1.zip › Figure 3-source data 2/Figure 3-source data 2 original files/Figure 3A Exo8.tif]

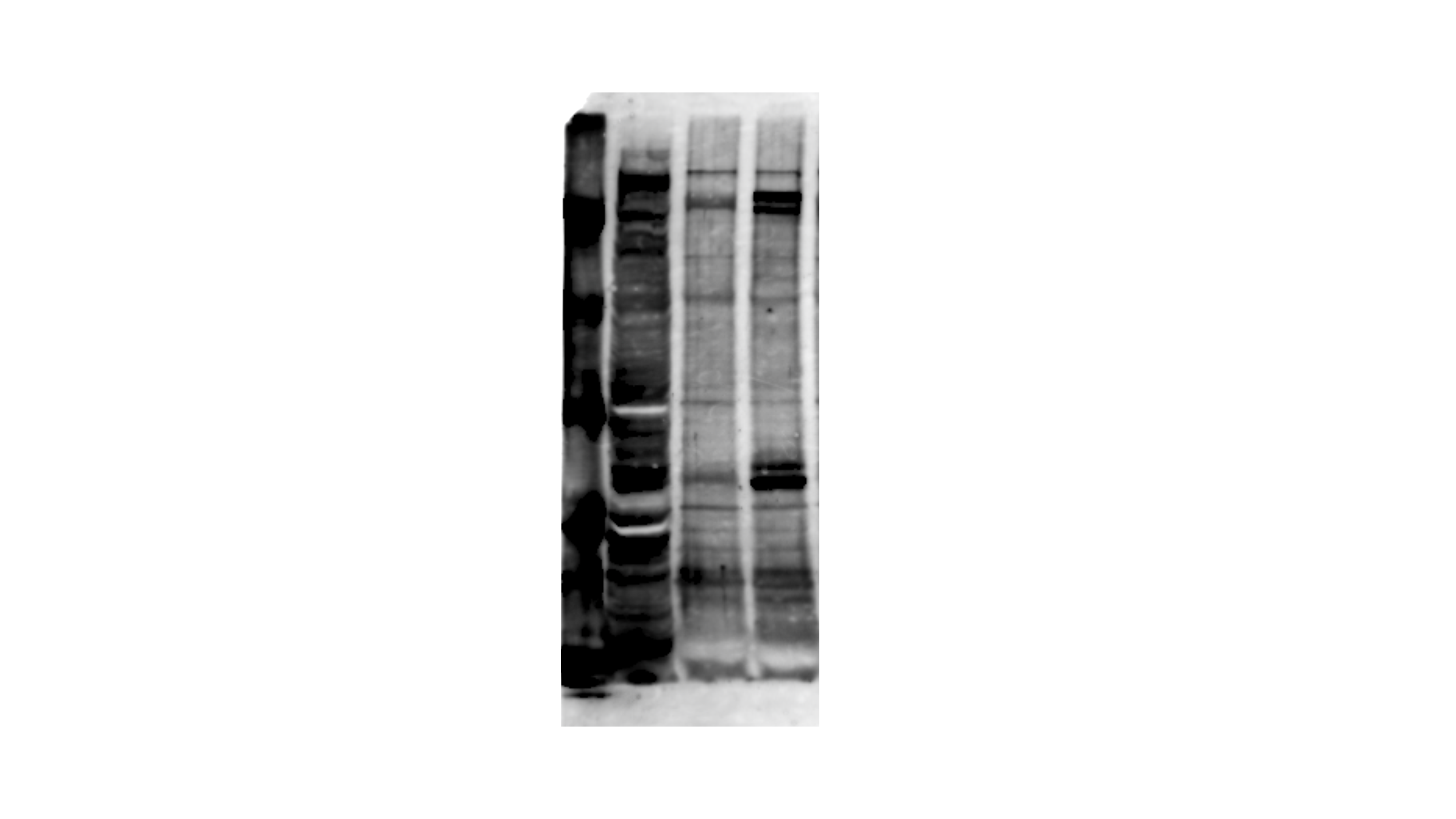

Supplement: Figure 3—source data 1. [file elife-82821-fig3-data1.zip › Figure 3-source data 2/Figure 3-source data 2 original files/Figure 3C Mlph.tif]

# Source data 3

## Uncropped blot images of figure supplement 1A

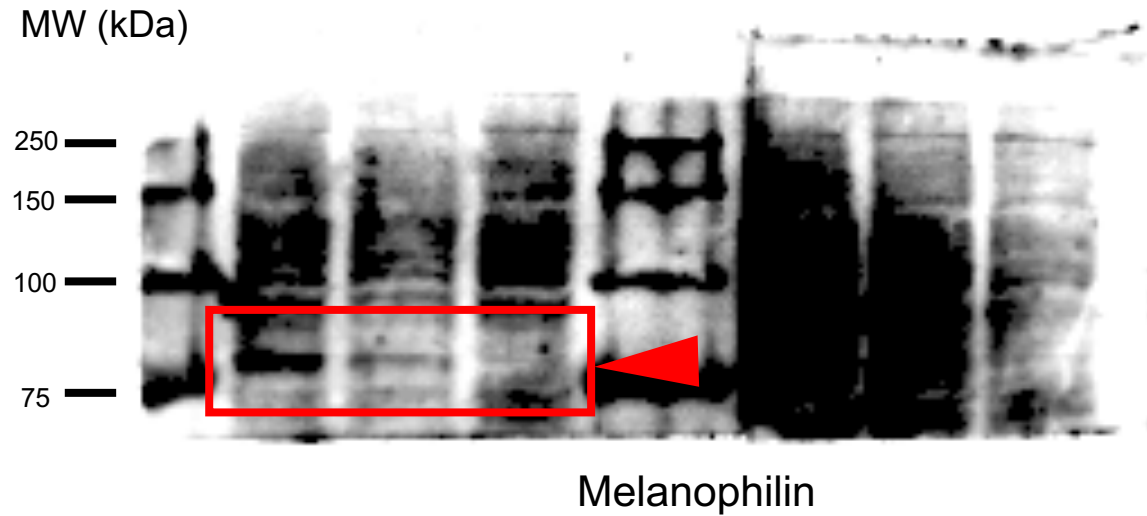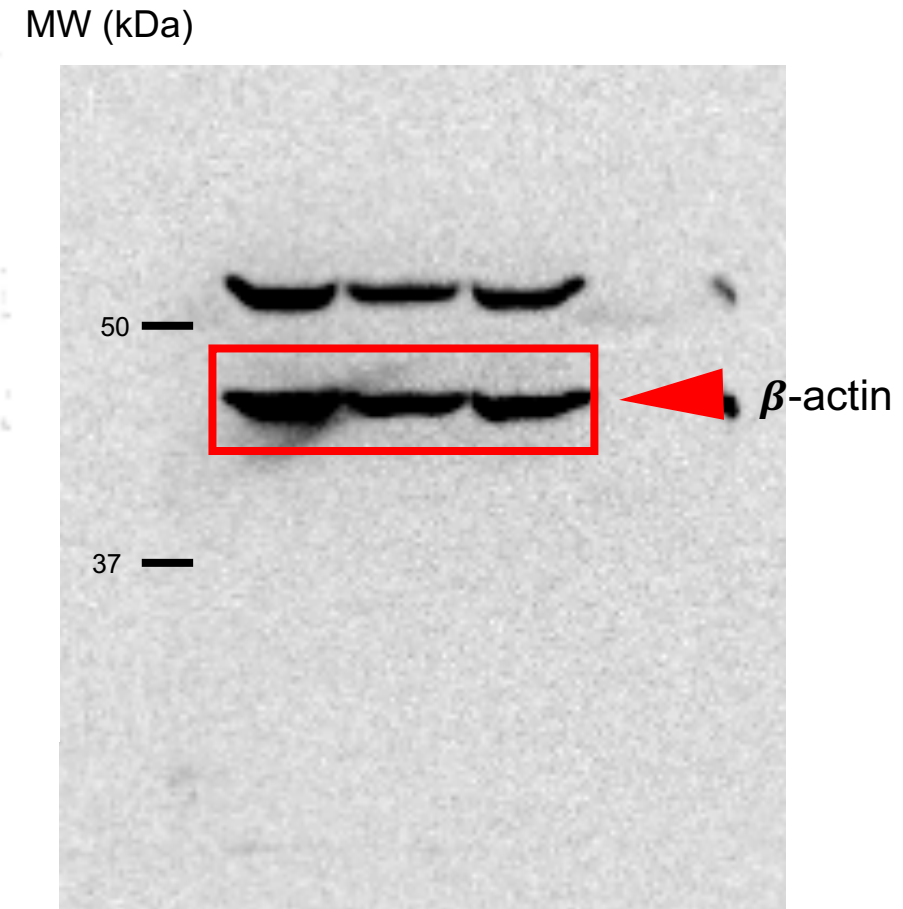

Supplement: Figure 3—figure supplement 1—source data 1. [file elife-82821-fig3-figsupp1-data1.zip › Figure 3-figure supplement 1-source data 3/Figure 3-figure supplement 1-source data 3.pdf]

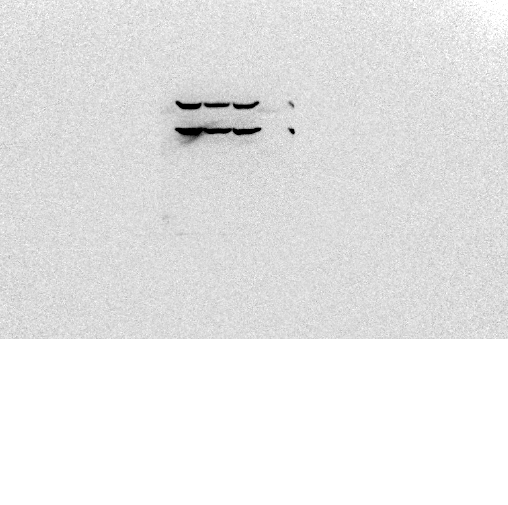

Supplement: Figure 3—figure supplement 1—source data 1. [file elife-82821-fig3-figsupp1-data1.zip › Figure 3-figure supplement 1-source data 3/Figure 3-figure supplement 1-source data 3 original files/figure supplement 1A actin.tif]

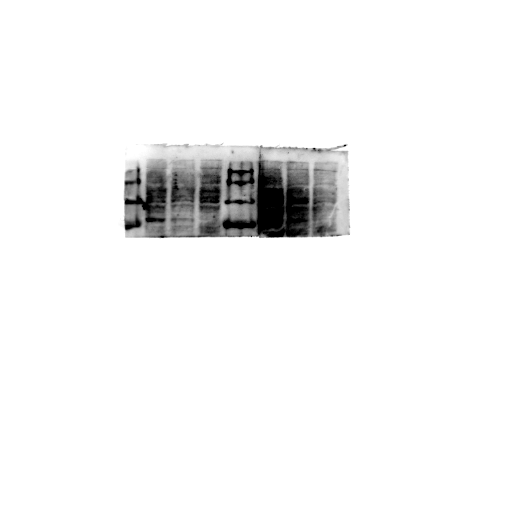

Supplement: Figure 3—figure supplement 1—source data 1. [file elife-82821-fig3-figsupp1-data1.zip › Figure 3-figure supplement 1-source data 3/Figure 3-figure supplement 1-source data 3 original files/figure supplement 1A Mlph.tif]

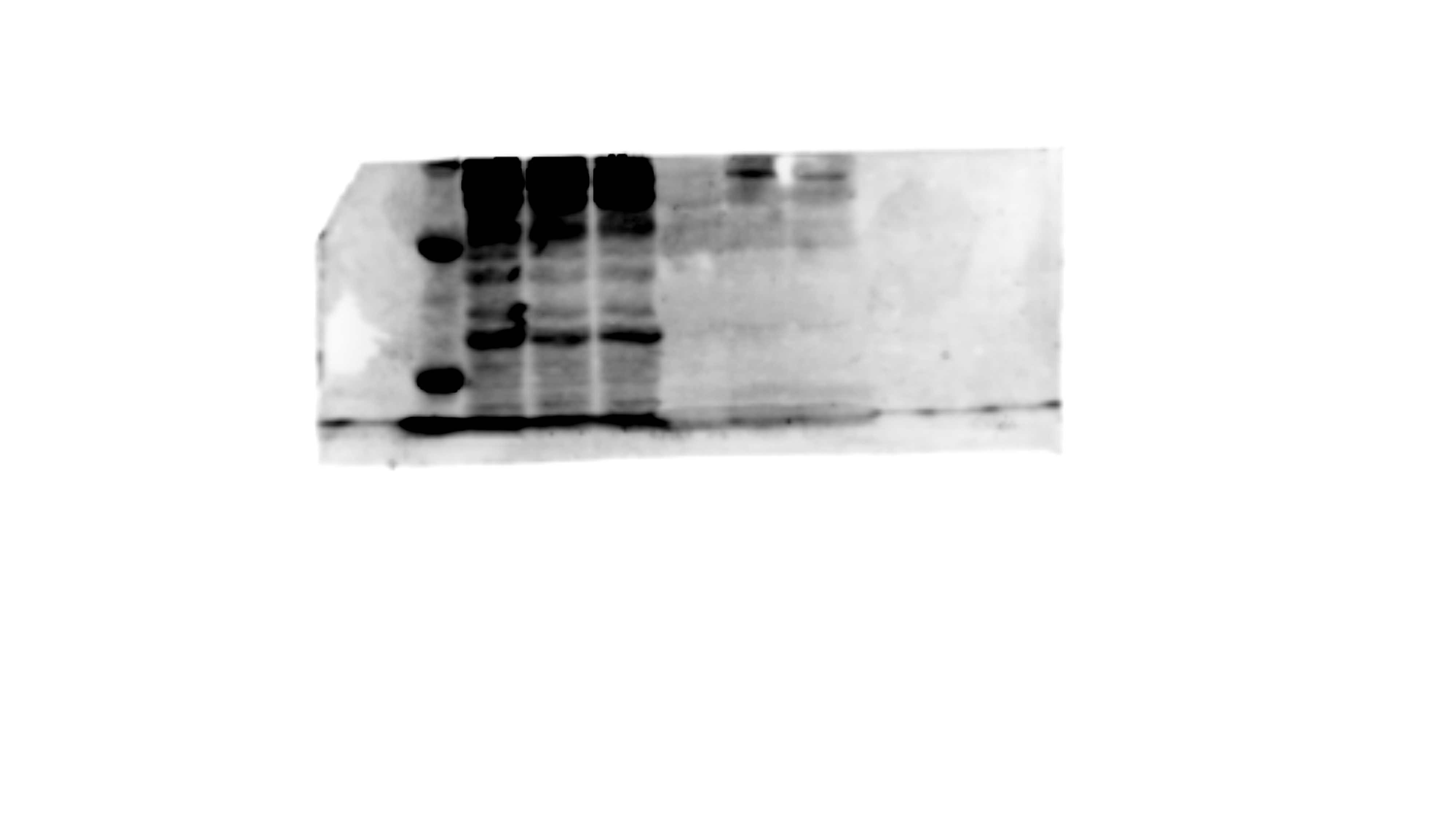

Supplement: Figure 3—figure supplement 2—source data 1. [file elife-82821-fig3-figsupp2-data1.zip › Figure 3-figure supplement 2-source data 4/Figure 3-figure supplement 2-source data 4 original files/figure supplement 2B Flag 4th.tif]

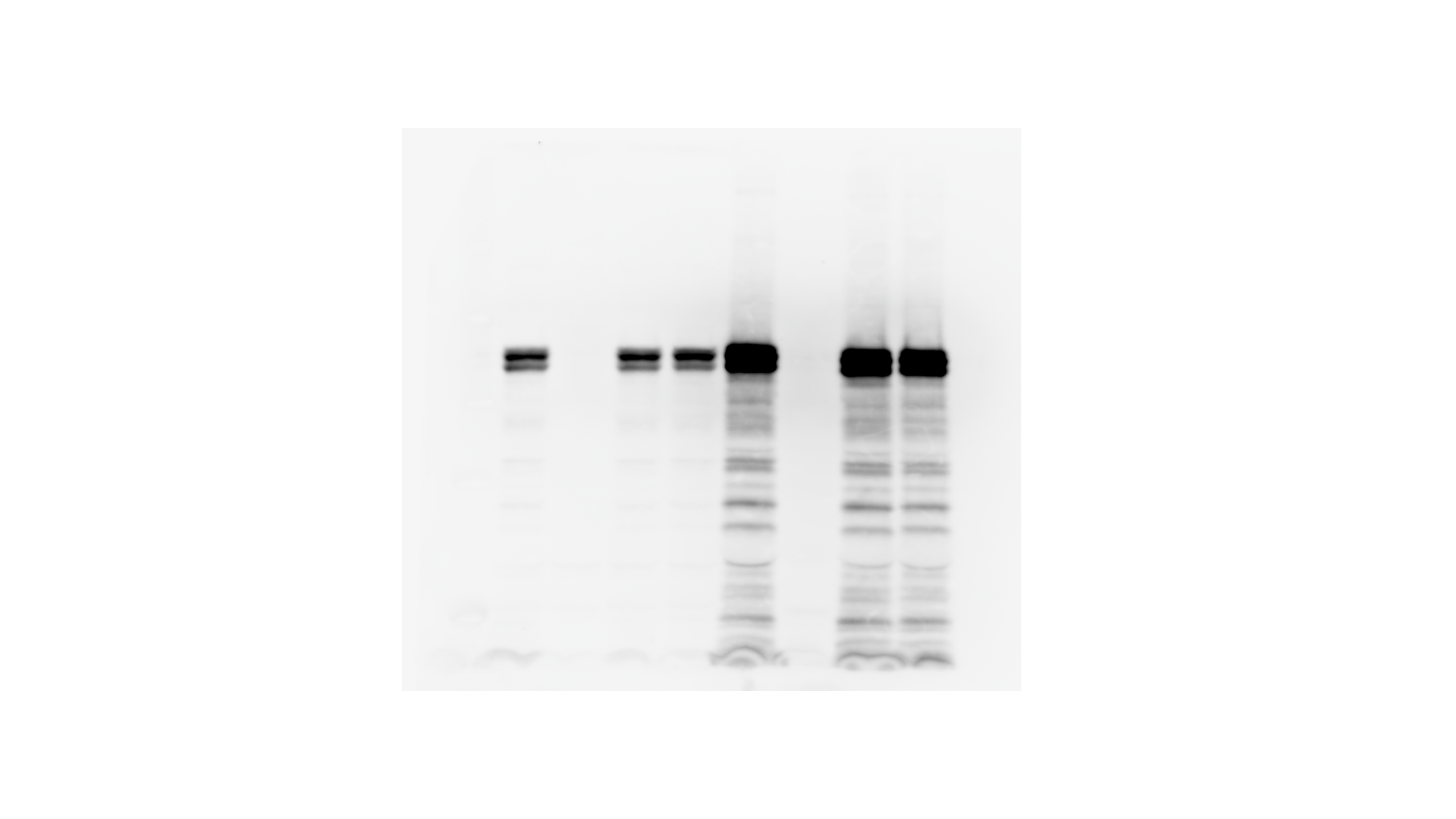

Supplement: Figure 3—figure supplement 2—source data 1. [file elife-82821-fig3-figsupp2-data1.zip › Figure 3-figure supplement 2-source data 4/Figure 3-figure supplement 2-source data 4 original files/figure supplement 2B RFP 2nd.tif]

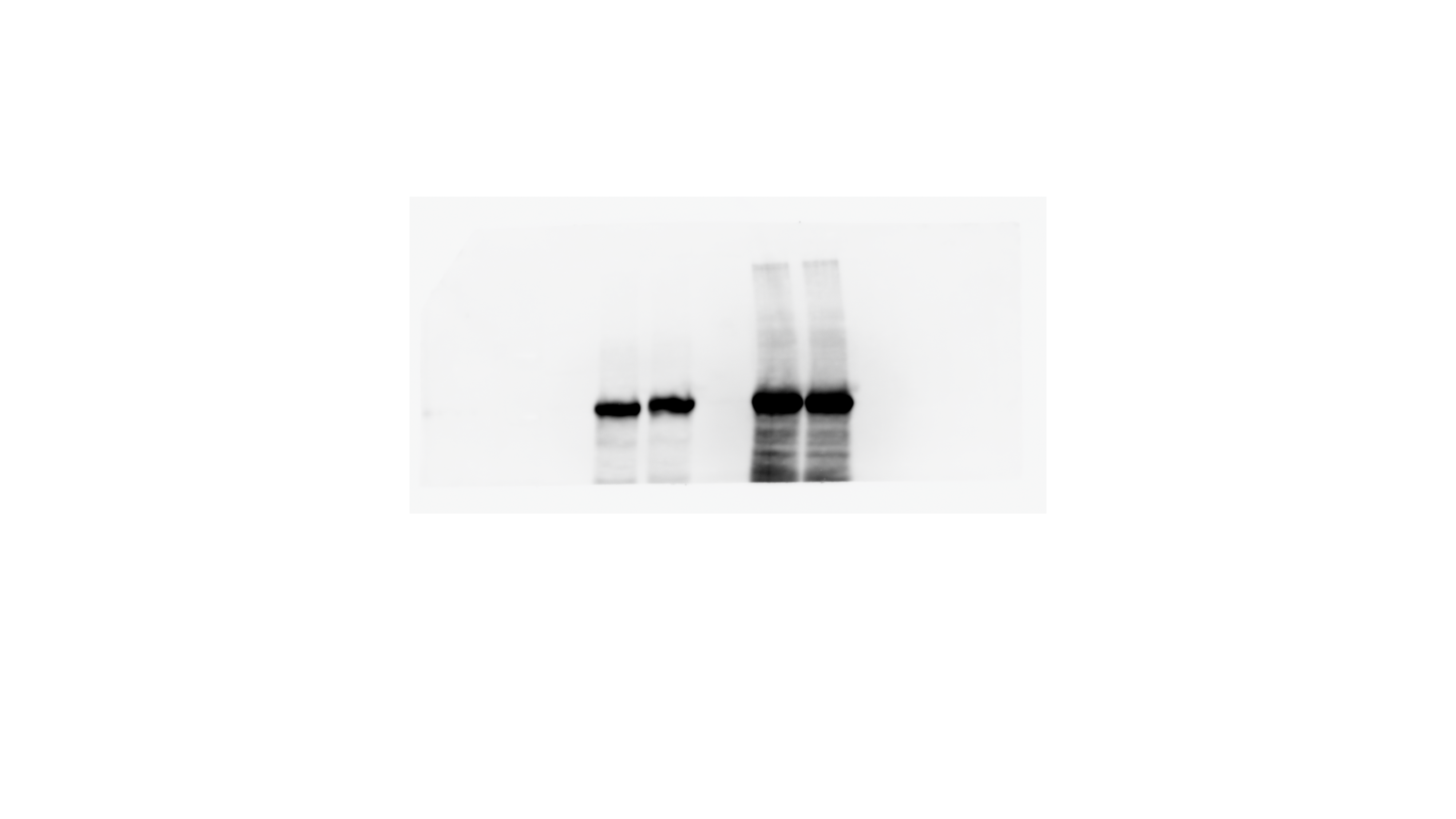

Supplement: Figure 3—figure supplement 2—source data 1. [file elife-82821-fig3-figsupp2-data1.zip › Figure 3-figure supplement 2-source data 4/Figure 3-figure supplement 2-source data 4 original files/figure supplement 2B RFP 4th.tif]

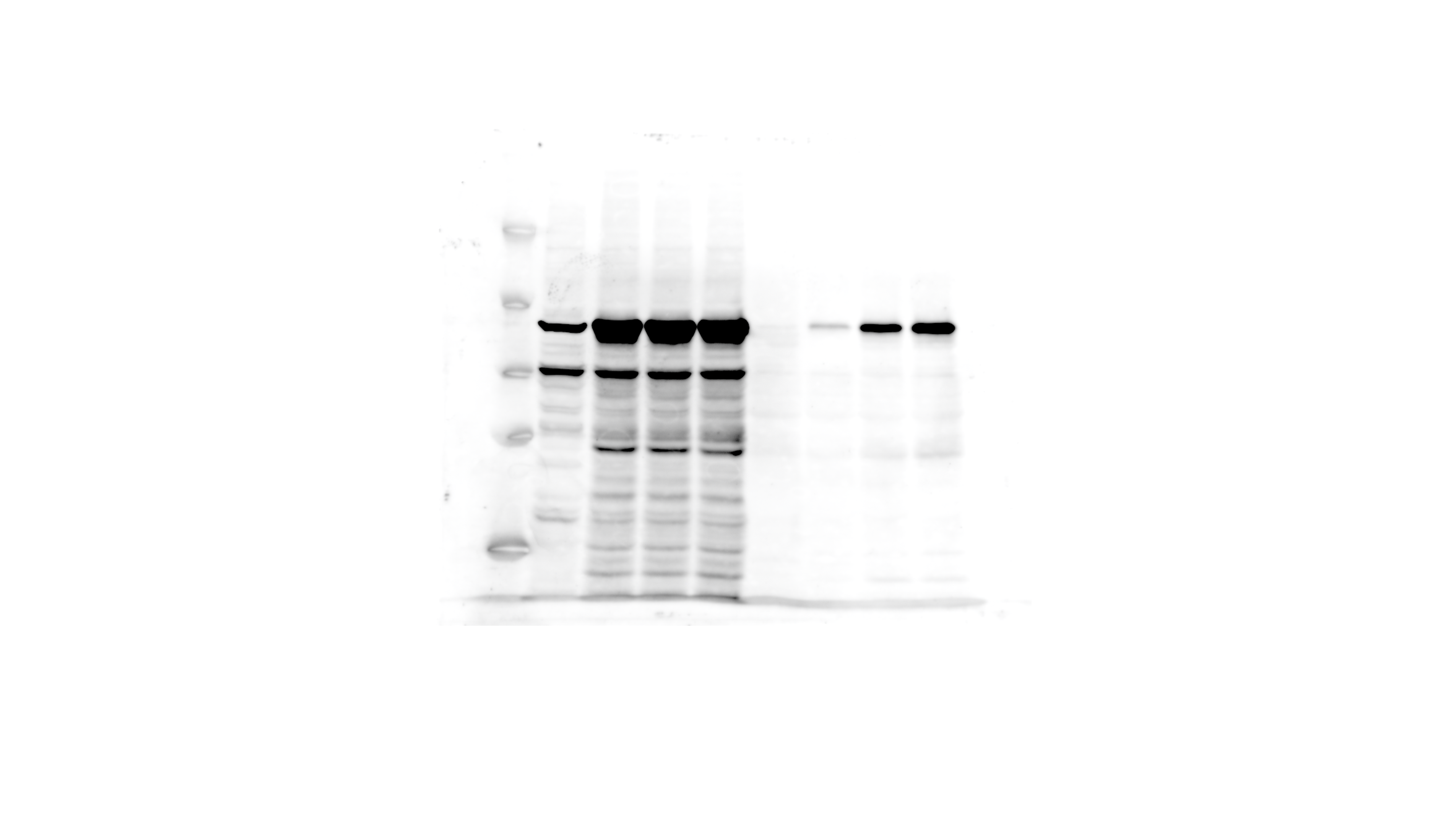

Supplement: Figure 3—figure supplement 2—source data 1. [file elife-82821-fig3-figsupp2-data1.zip › Figure 3-figure supplement 2-source data 4/Figure 3-figure supplement 2-source data 4 original files/figure supplement 2B Flag 2nd.tif]

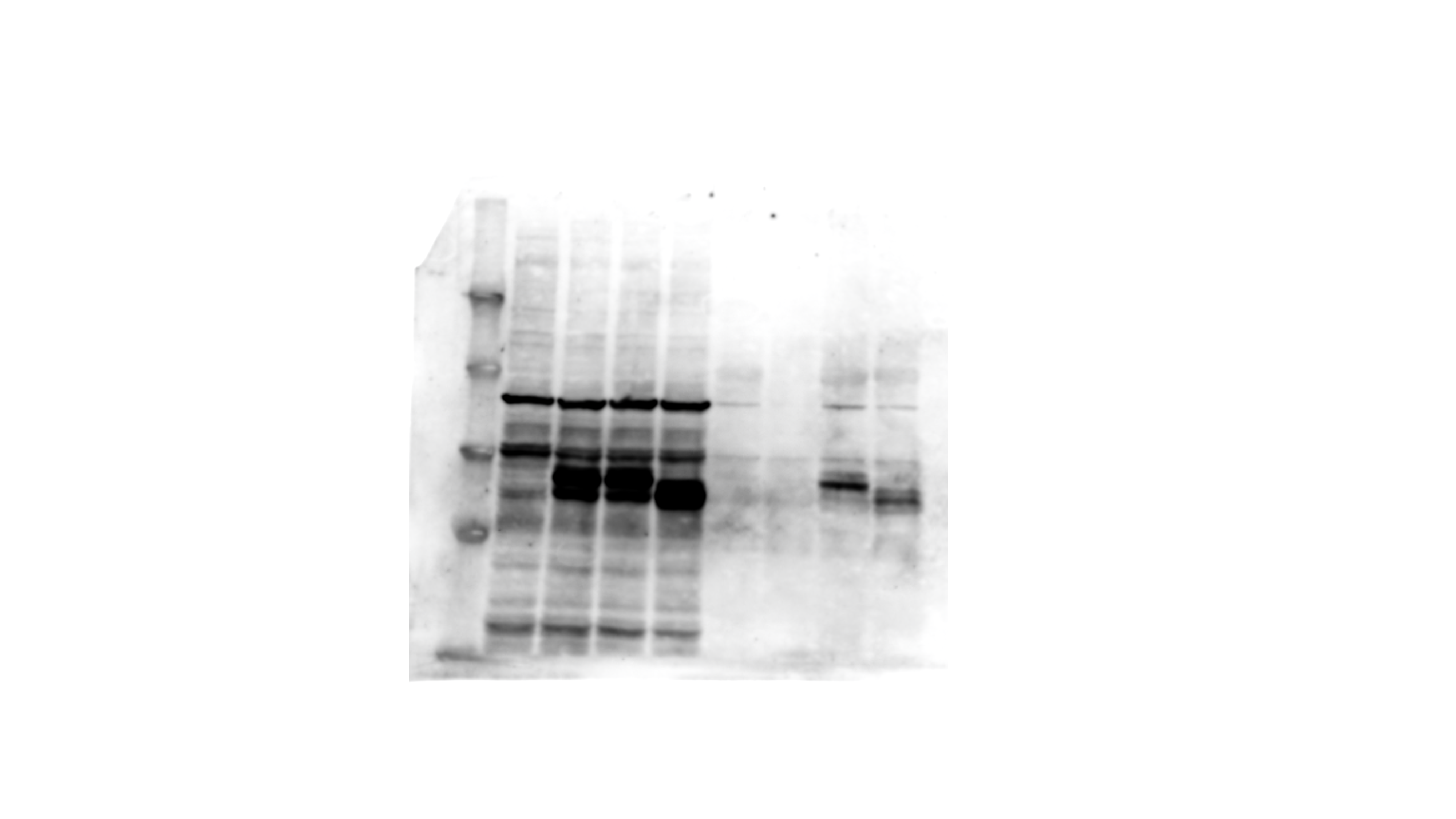

Supplement: Figure 3—figure supplement 2—source data 1. [file elife-82821-fig3-figsupp2-data1.zip › Figure 3-figure supplement 2-source data 4/Figure 3-figure supplement 2-source data 4 original files/figure supplement 2B Flag 3rd.tif]

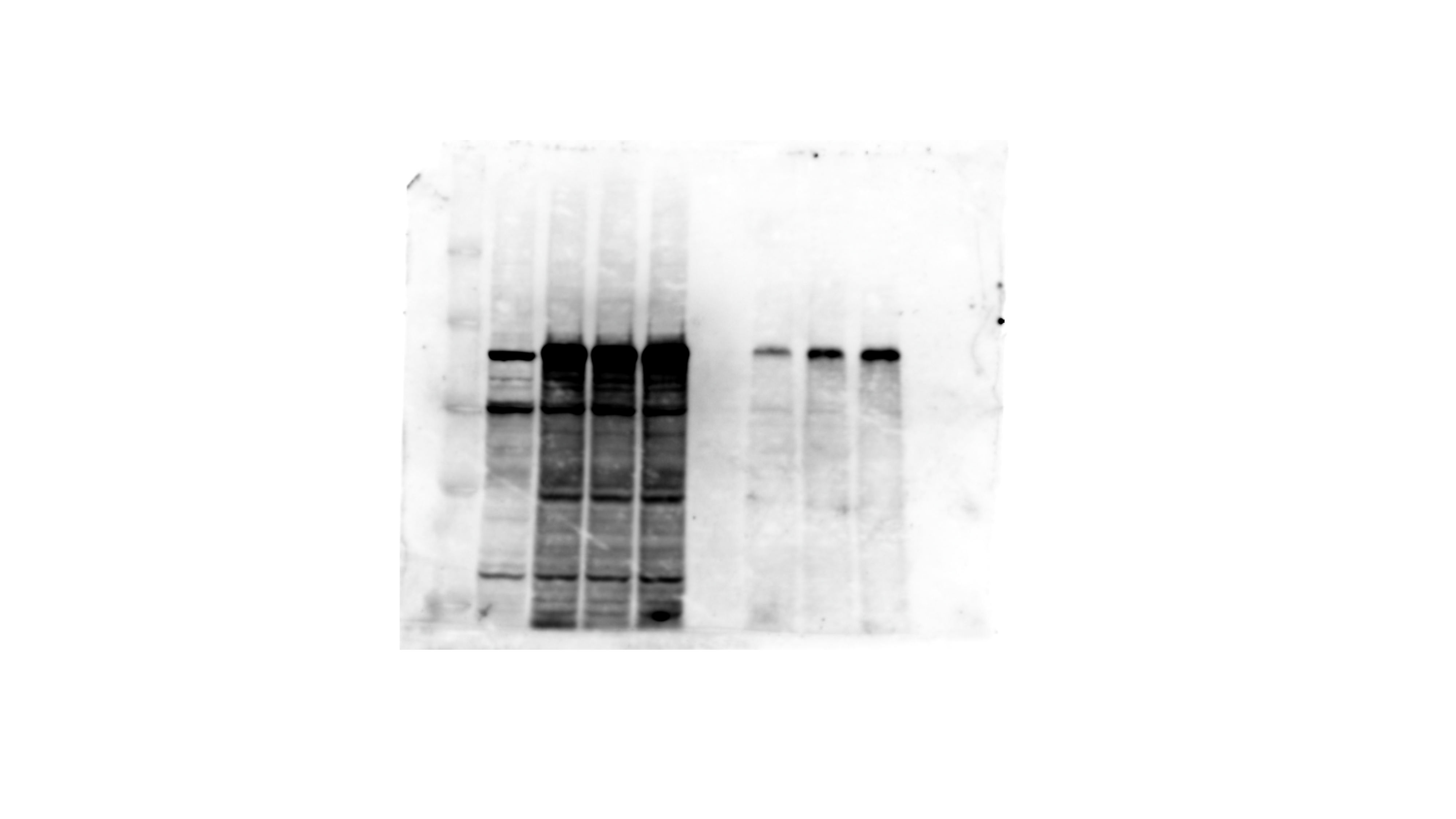

Supplement: Figure 3—figure supplement 2—source data 1. [file elife-82821-fig3-figsupp2-data1.zip › Figure 3-figure supplement 2-source data 4/Figure 3-figure supplement 2-source data 4 original files/figure supplement 2B Flag 1st.tif]

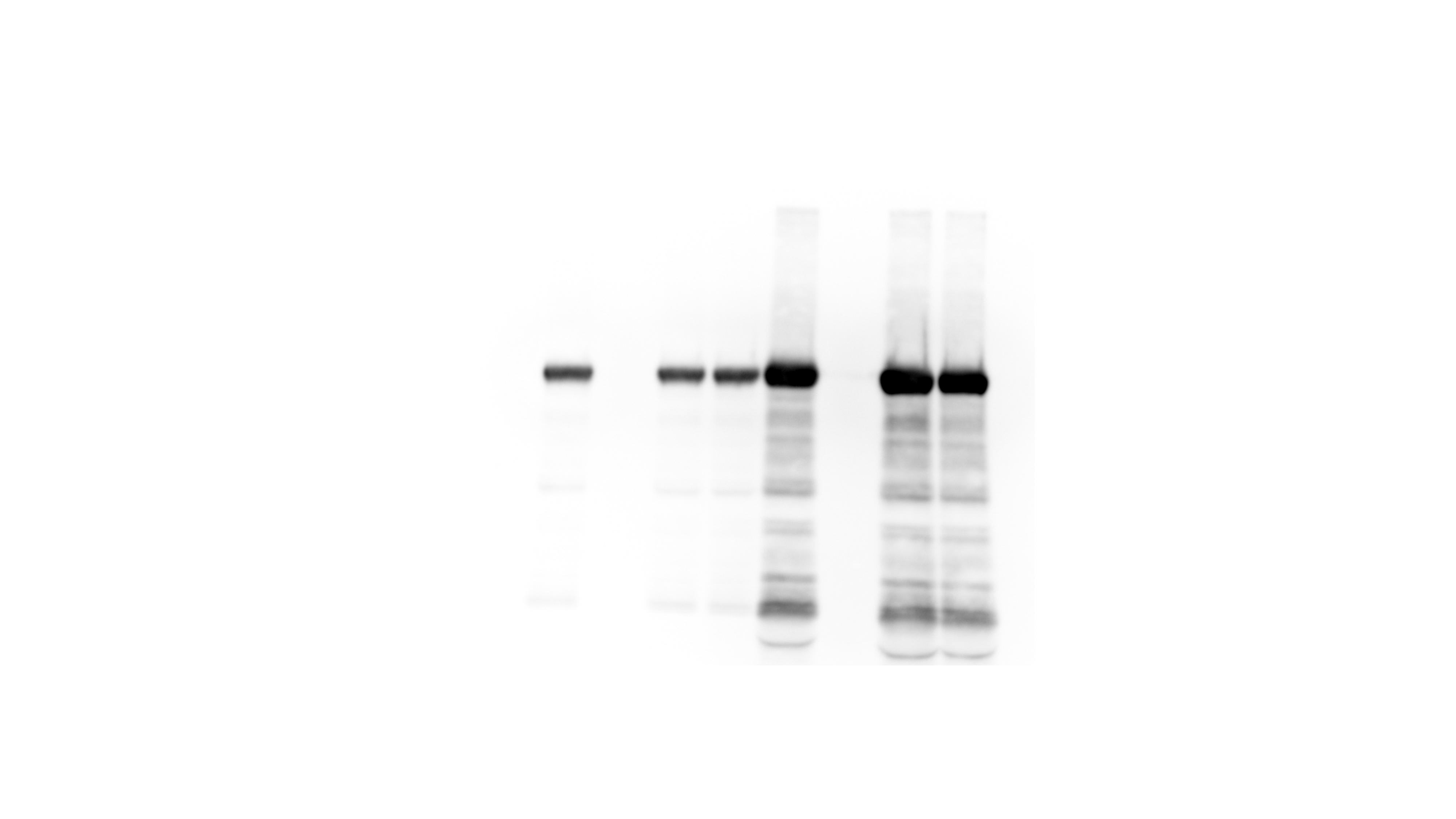

Supplement: Figure 3—figure supplement 2—source data 1. [file elife-82821-fig3-figsupp2-data1.zip › Figure 3-figure supplement 2-source data 4/Figure 3-figure supplement 2-source data 4 original files/figure supplement 2B RFP 3rd.tif]

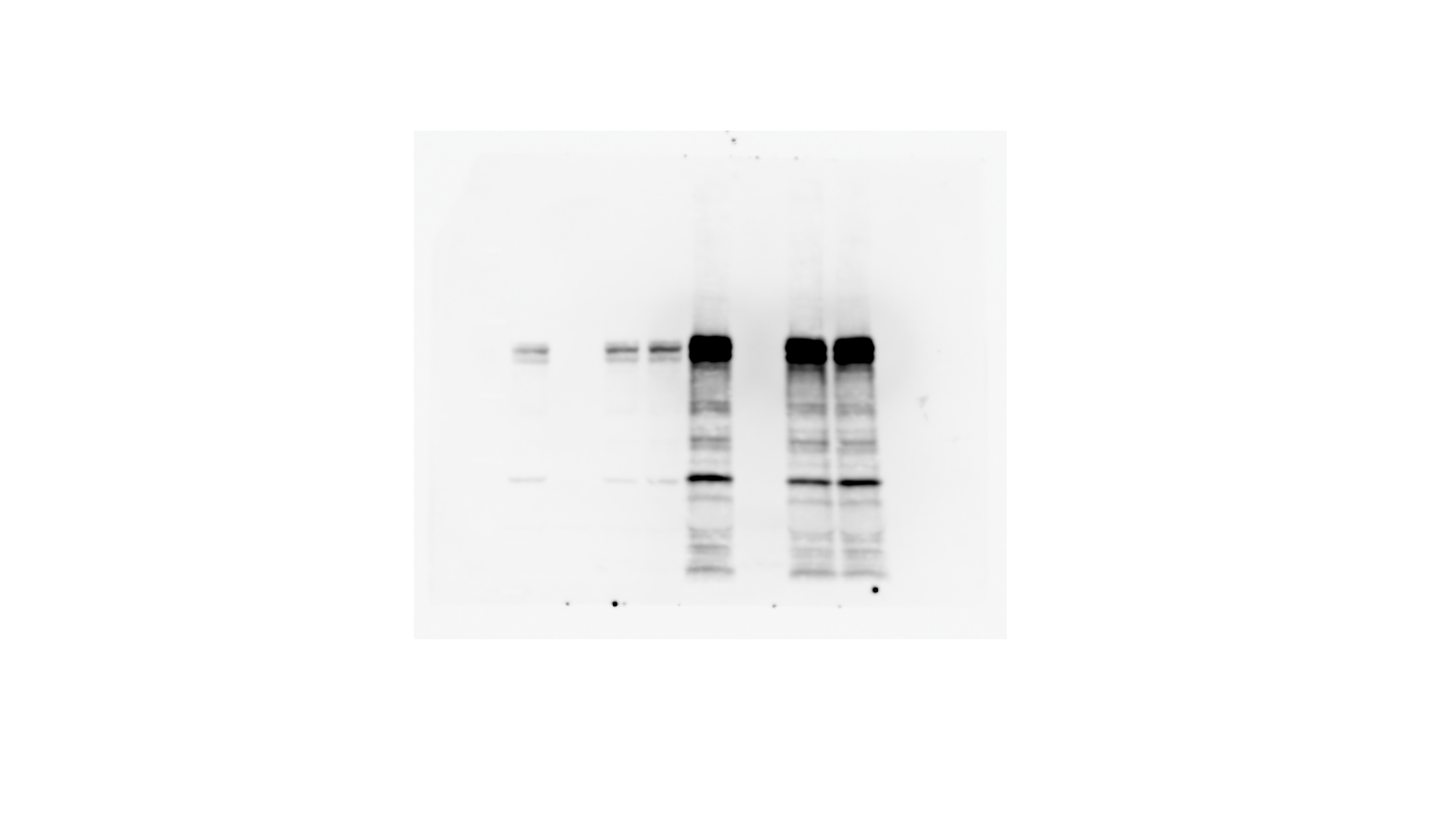

Supplement: Figure 3—figure supplement 2—source data 1. [file elife-82821-fig3-figsupp2-data1.zip › Figure 3-figure supplement 2-source data 4/Figure 3-figure supplement 2-source data 4 original files/figure supplement 2B RFP 1st.tif]

# Source data 5

## Uncropped blot images of Figure 4B

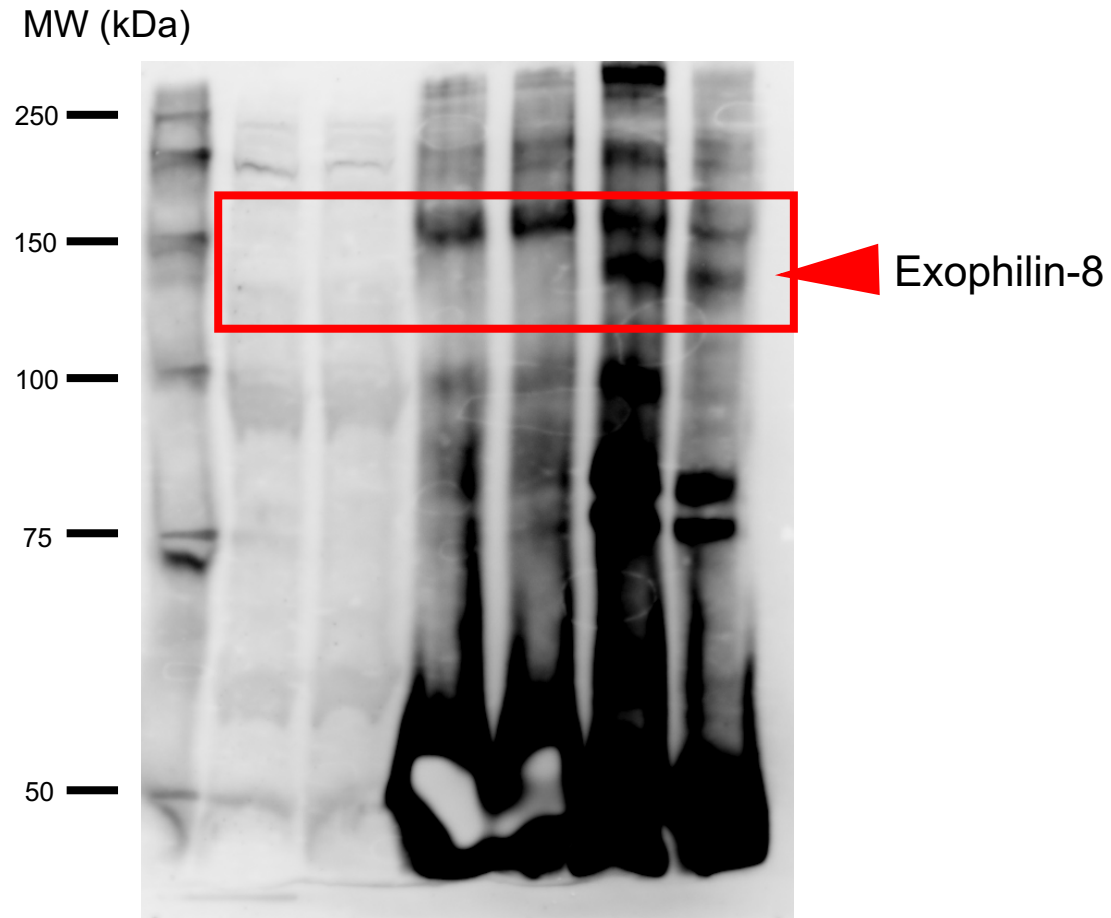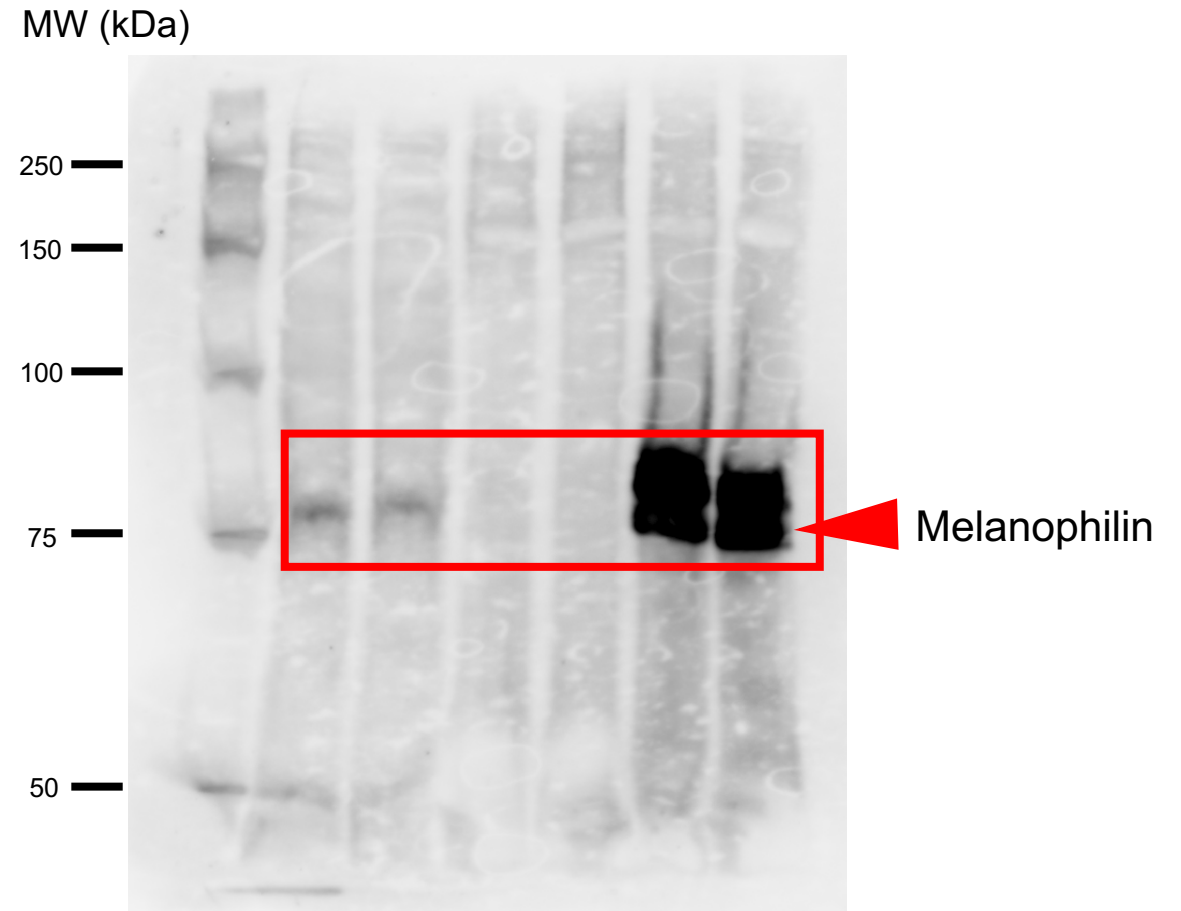

Supplement: Figure 4—source data 1. [file elife-82821-fig4-data1.zip › Figure 4-source data 5/Figure 4-source data 5.pdf]

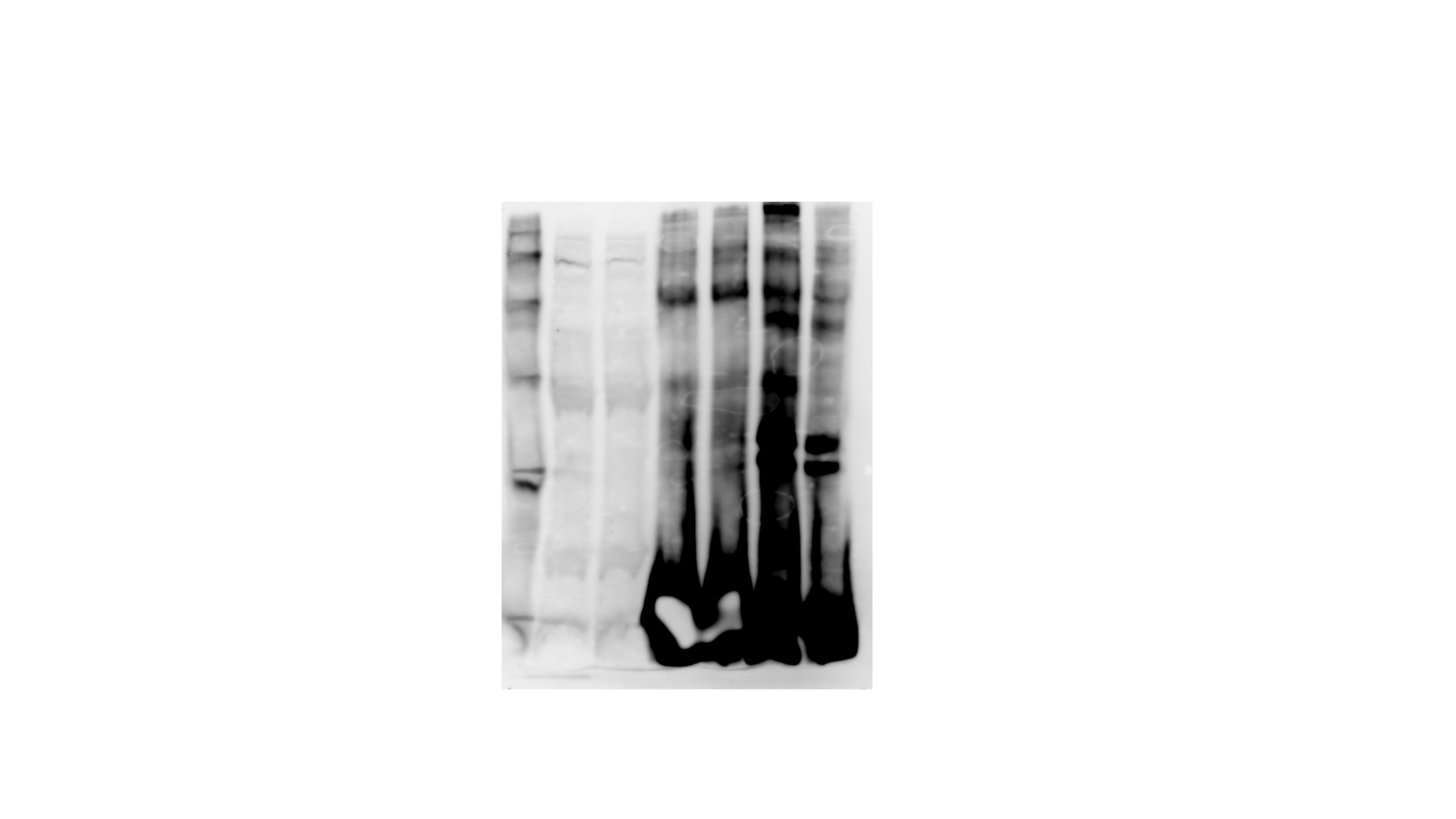

Supplement: Figure 4—source data 1. [file elife-82821-fig4-data1.zip › Figure 4-source data 5/Figure 4-source data 5 original files/Figure 4B Exo8.tif]

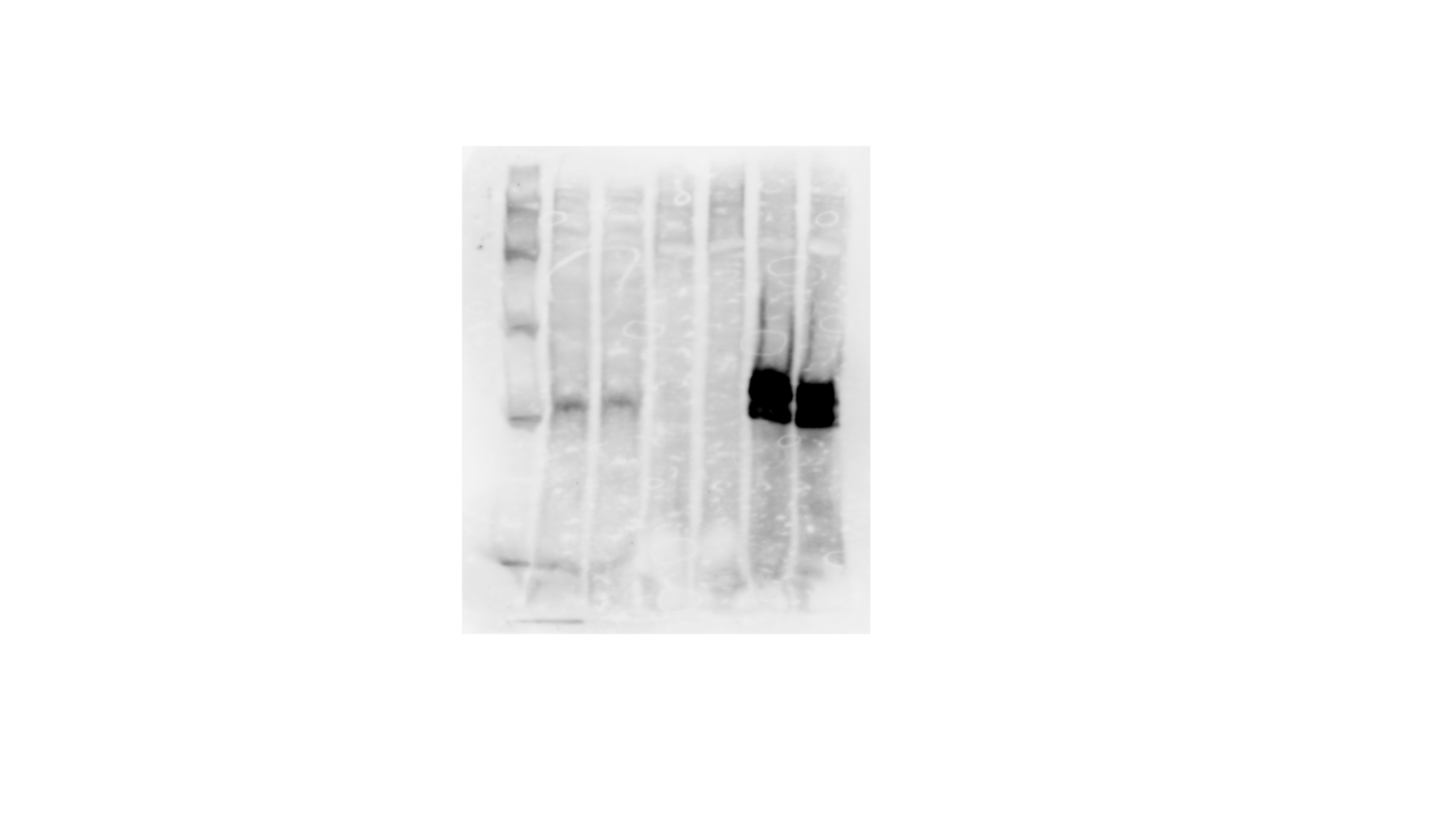

Supplement: Figure 4—source data 1. [file elife-82821-fig4-data1.zip › Figure 4-source data 5/Figure 4-source data 5 original files/Figure 4B Mlph.tif]

# Source data 6

## Uncropped blot images of figure supplement 1A

(left)

MW (kDa)

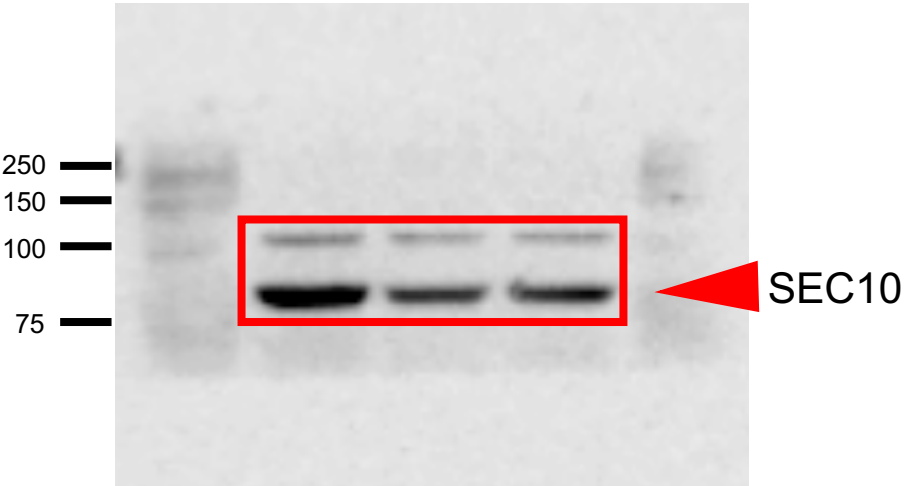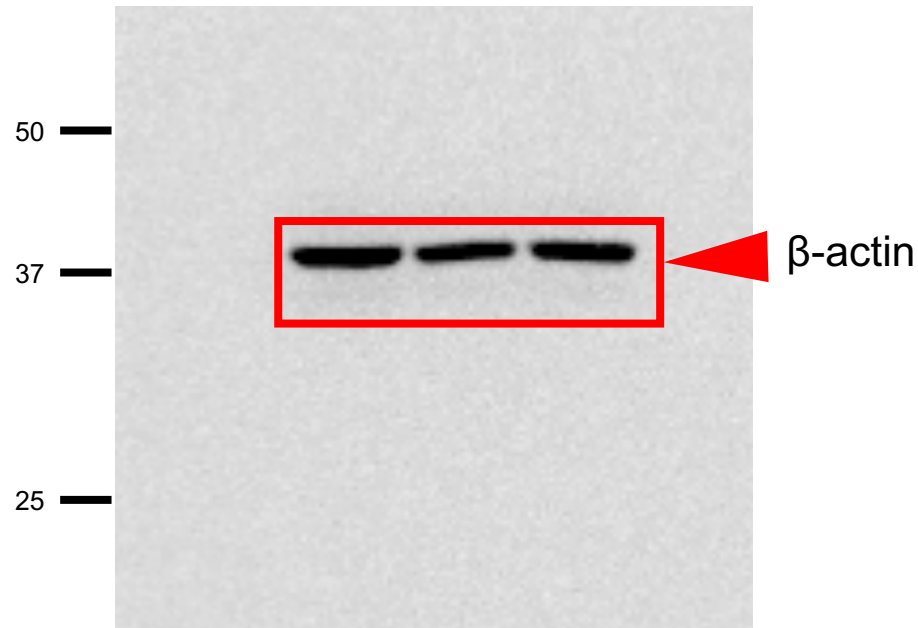

(right)

MW (kDa)

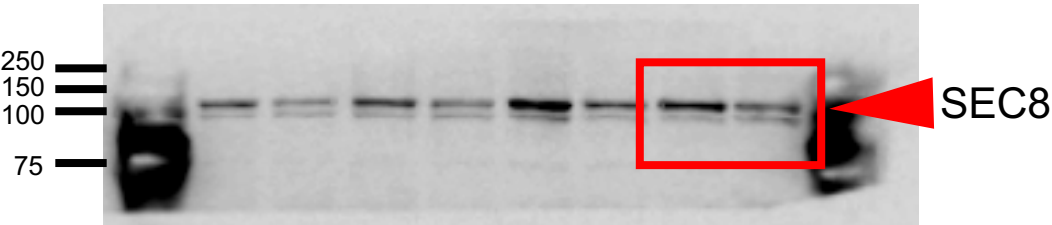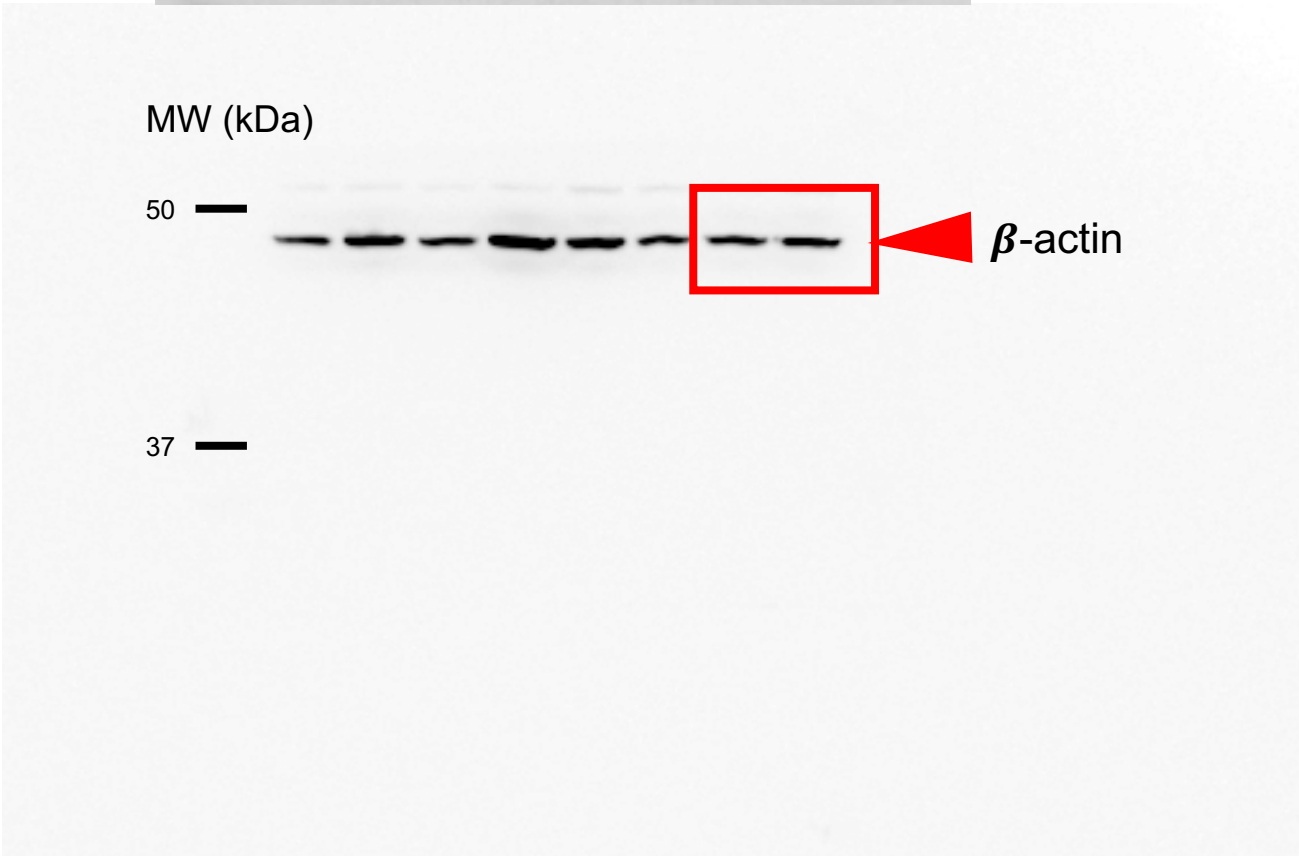

Supplement: Figure 4—figure supplement 1—source data 1. [file elife-82821-fig4-figsupp1-data1.zip › Figure 4-figure supplement 1-source data 6/Figure 4-figure supplement 1-source data 6.pdf]

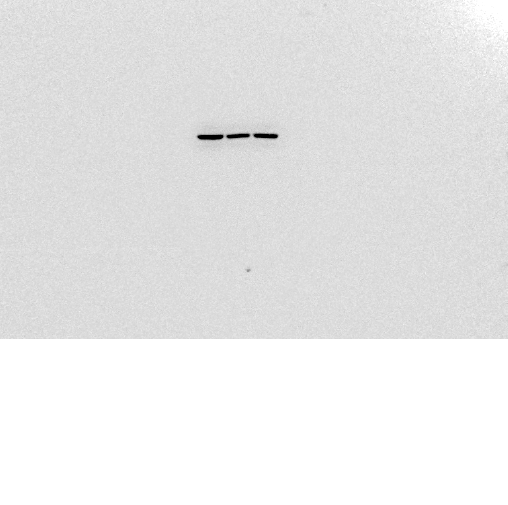

Supplement: Figure 4—figure supplement 1—source data 1. [file elife-82821-fig4-figsupp1-data1.zip › Figure 4-figure supplement 1-source data 6/Figure 4-figure supplement 1-source data 6 original files/figure supplement 1A actin 1st.tif]

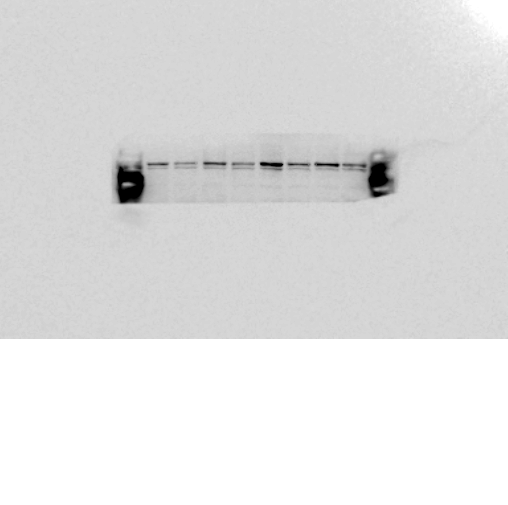

Supplement: Figure 4—figure supplement 1—source data 1. [file elife-82821-fig4-figsupp1-data1.zip › Figure 4-figure supplement 1-source data 6/Figure 4-figure supplement 1-source data 6 original files/figure supplement 1A SEC8KD.tif]

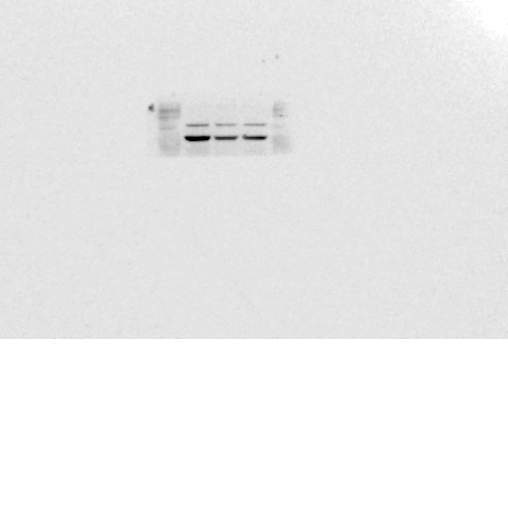

Supplement: Figure 4—figure supplement 1—source data 1. [file elife-82821-fig4-figsupp1-data1.zip › Figure 4-figure supplement 1-source data 6/Figure 4-figure supplement 1-source data 6 original files/figure supplement 1A SEC10KD.tif]

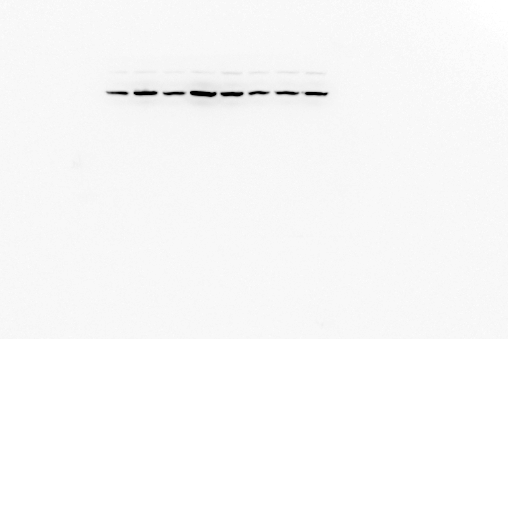

Supplement: Figure 4—figure supplement 1—source data 1. [file elife-82821-fig4-figsupp1-data1.zip › Figure 4-figure supplement 1-source data 6/Figure 4-figure supplement 1-source data 6 original files/figure supplement 1A actin 2nd.tif]
