# Supplementary material for: Functional hierarchy among different Rab27 effectors involved in secretory granule exocytosis
Source: eLife. 2023 Feb 21;12:e82821. doi: 10.7554/eLife.82821 (PMC9988257; doi:10.7554/eLife.82821)

# Source data 2

## Uncropped blot images of Figure 3A

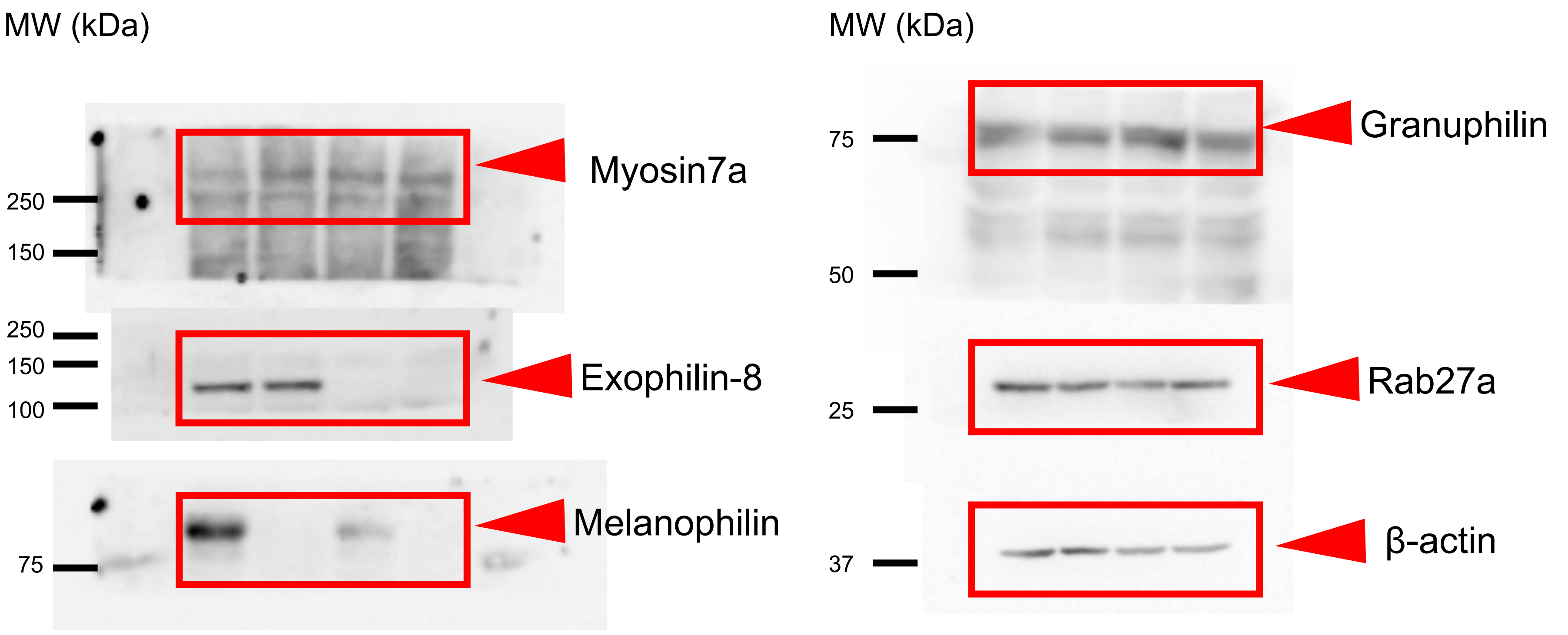

# Source data 2

## Uncropped blot images of Figure 3B

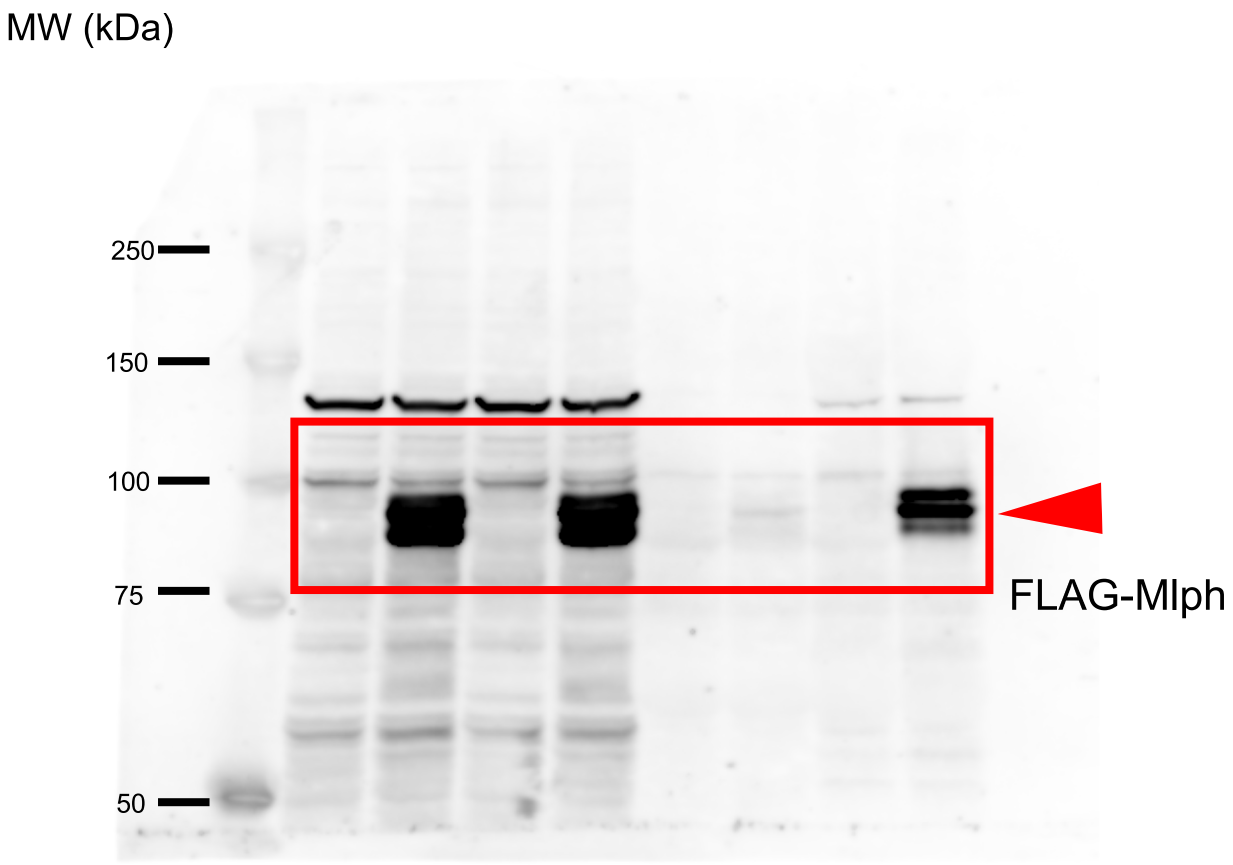


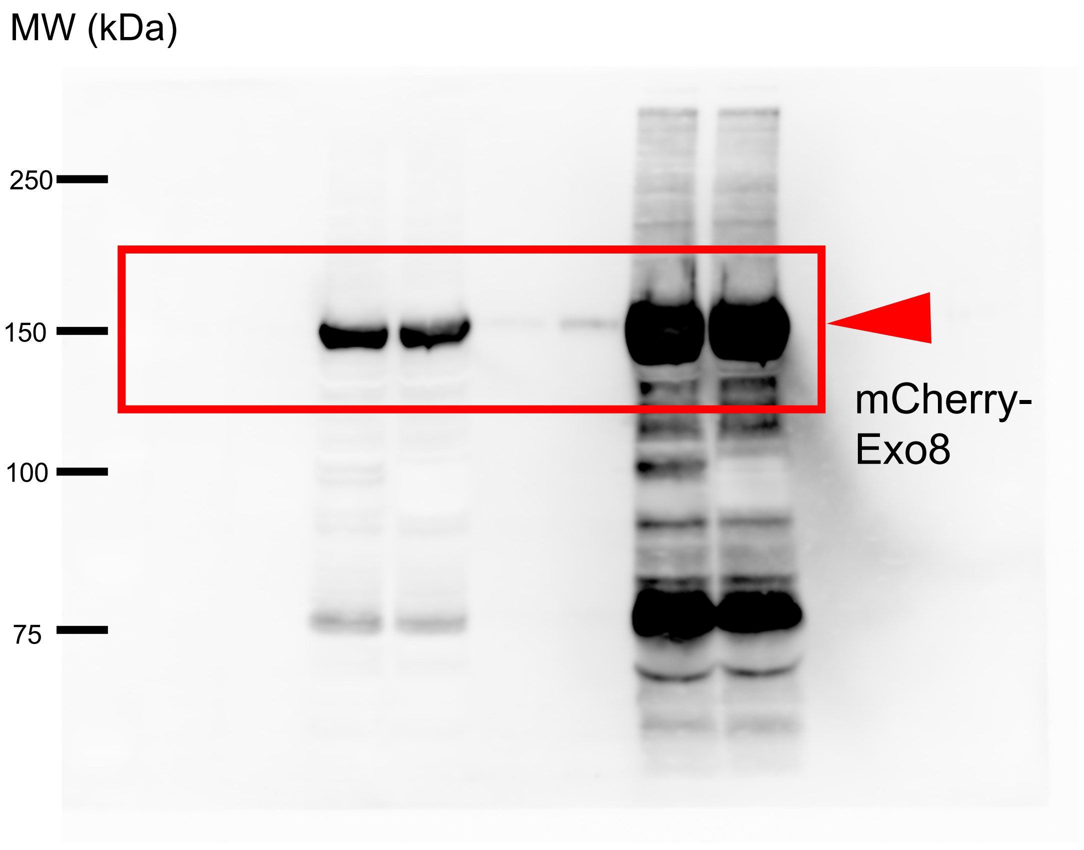

# Source data 2

## Uncropped blot images of Figure 3C

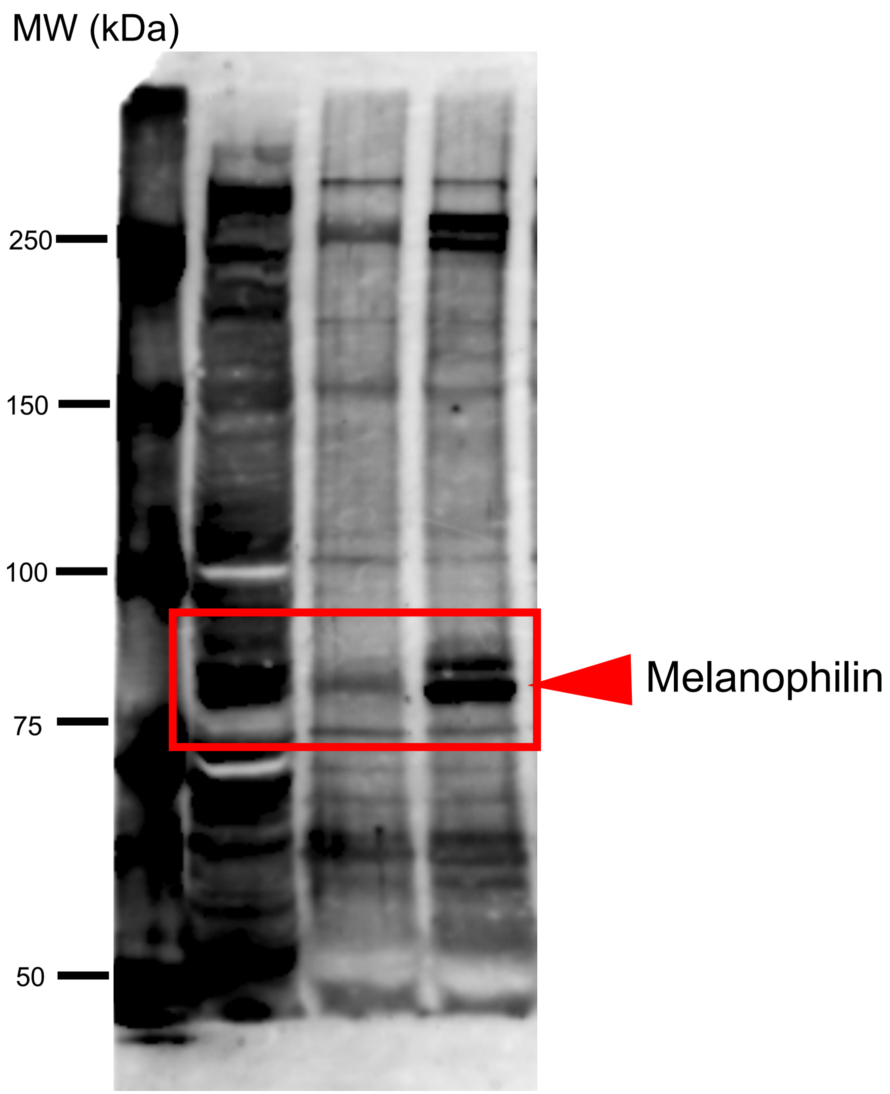


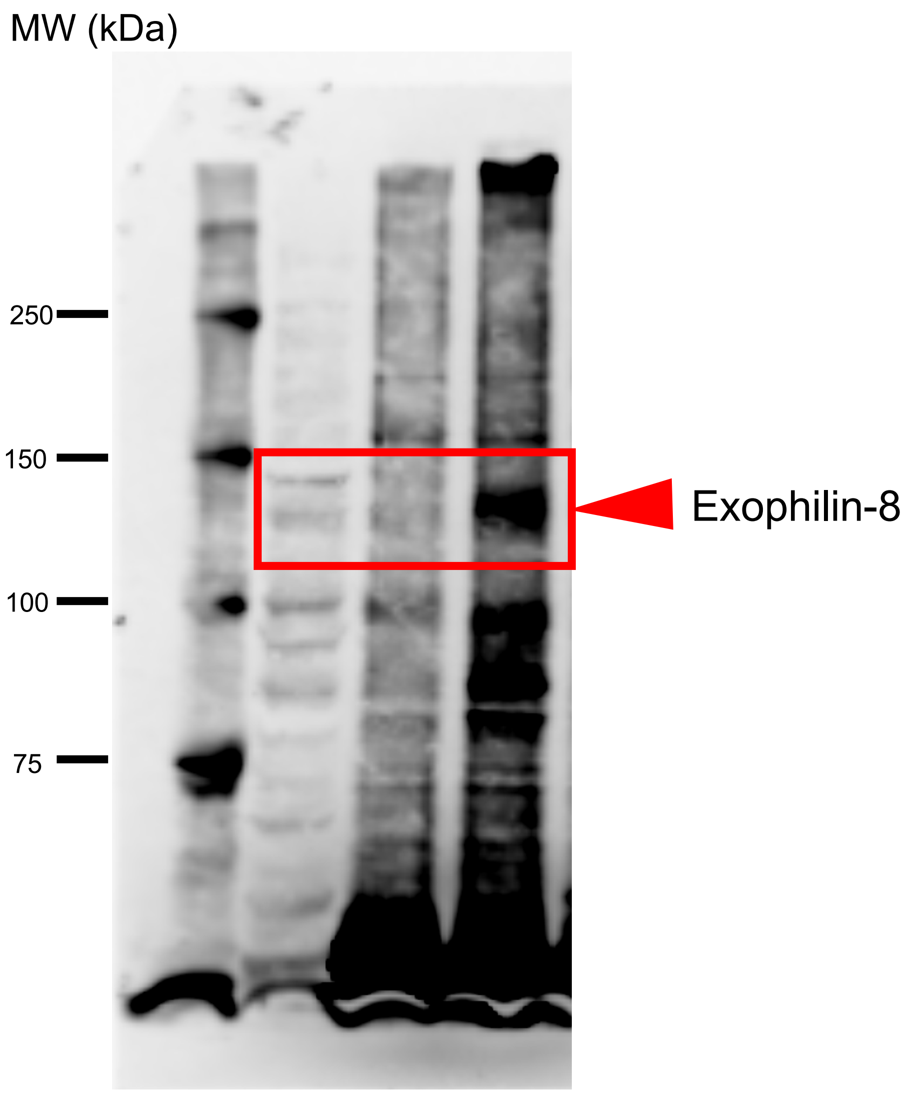

# Source data 2

## Uncropped blot images of Figure 3C

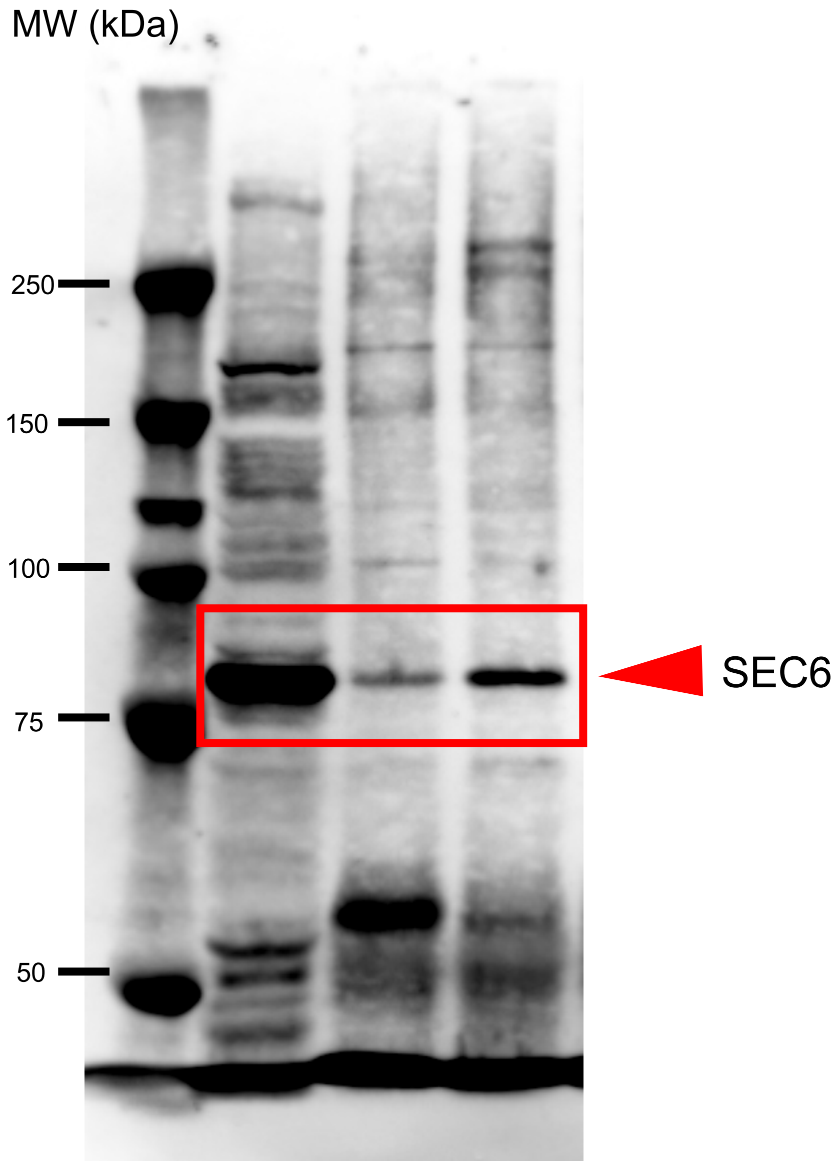


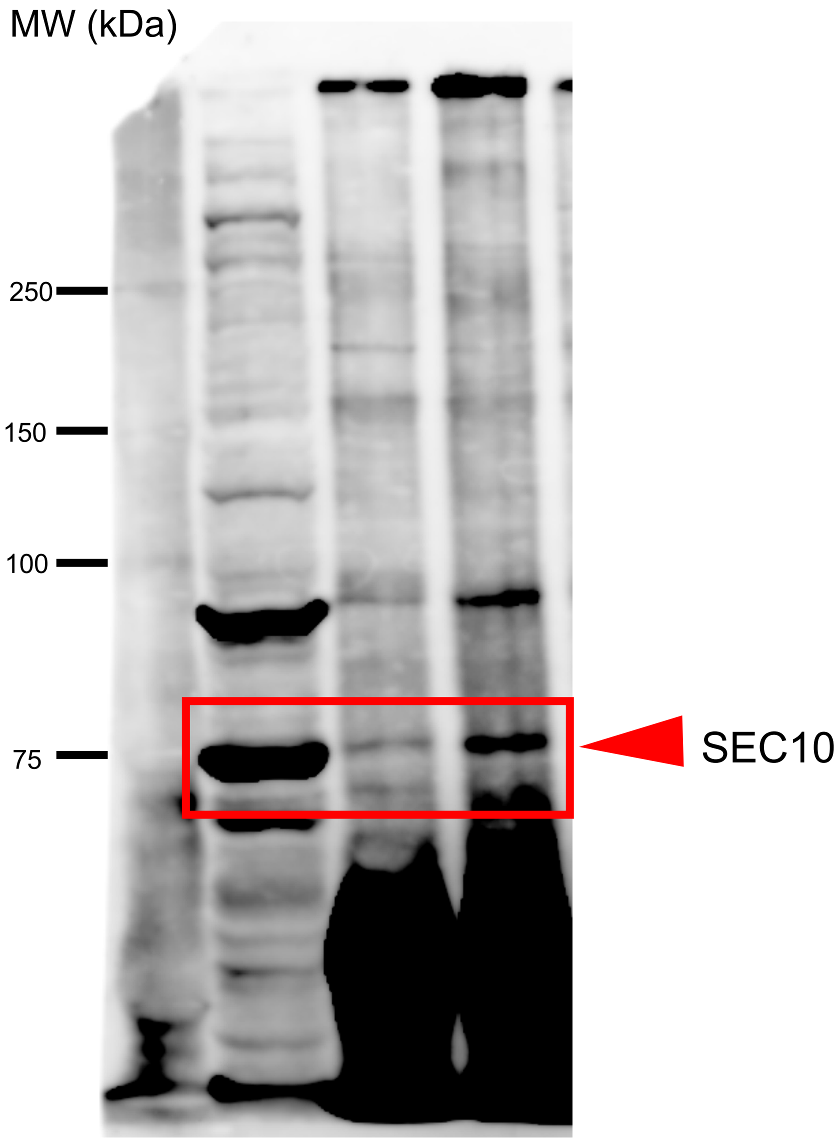

# Source data 2

## Uncropped blot images of Figure 3E

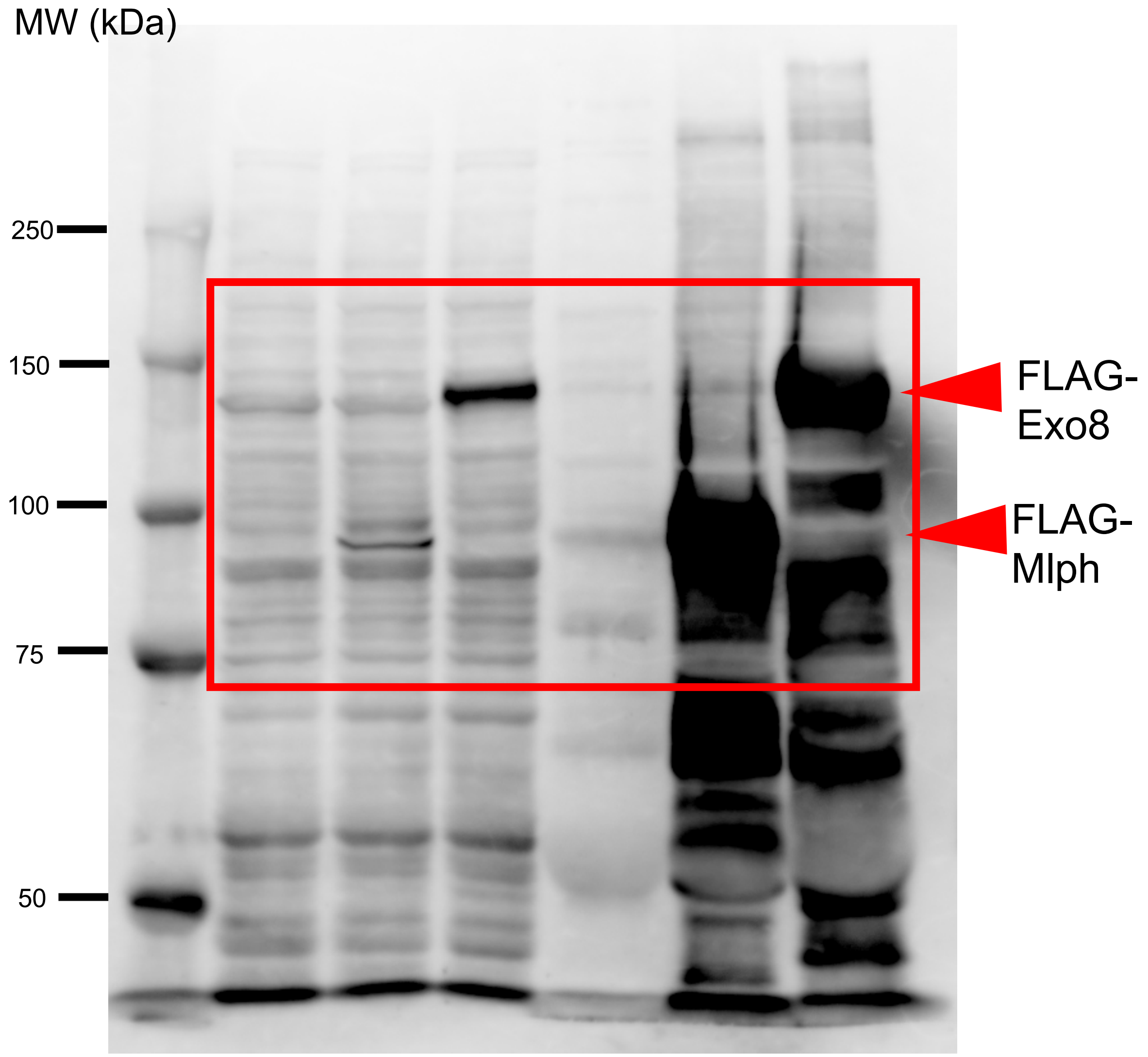


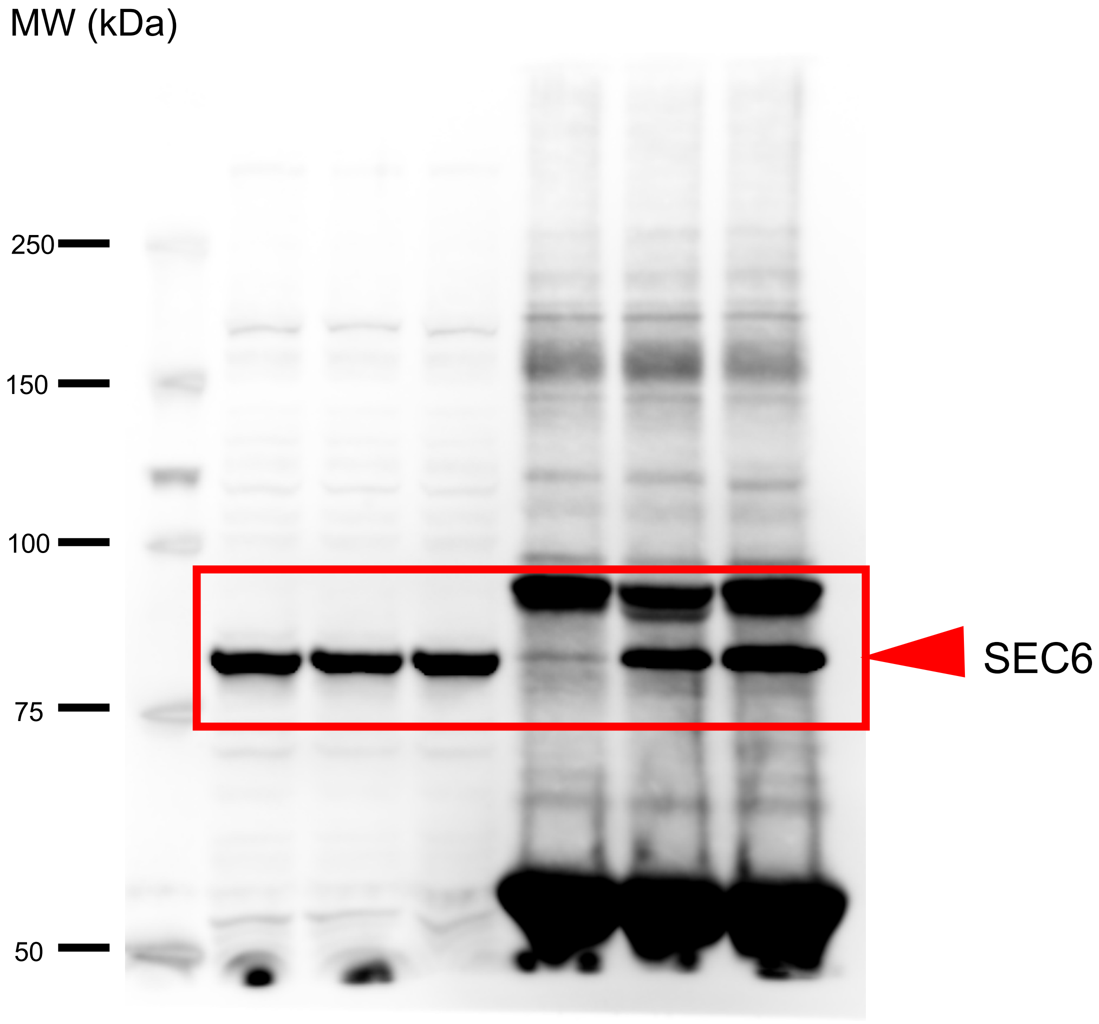

# Source data 2

## Uncropped blot images of Figure 3E

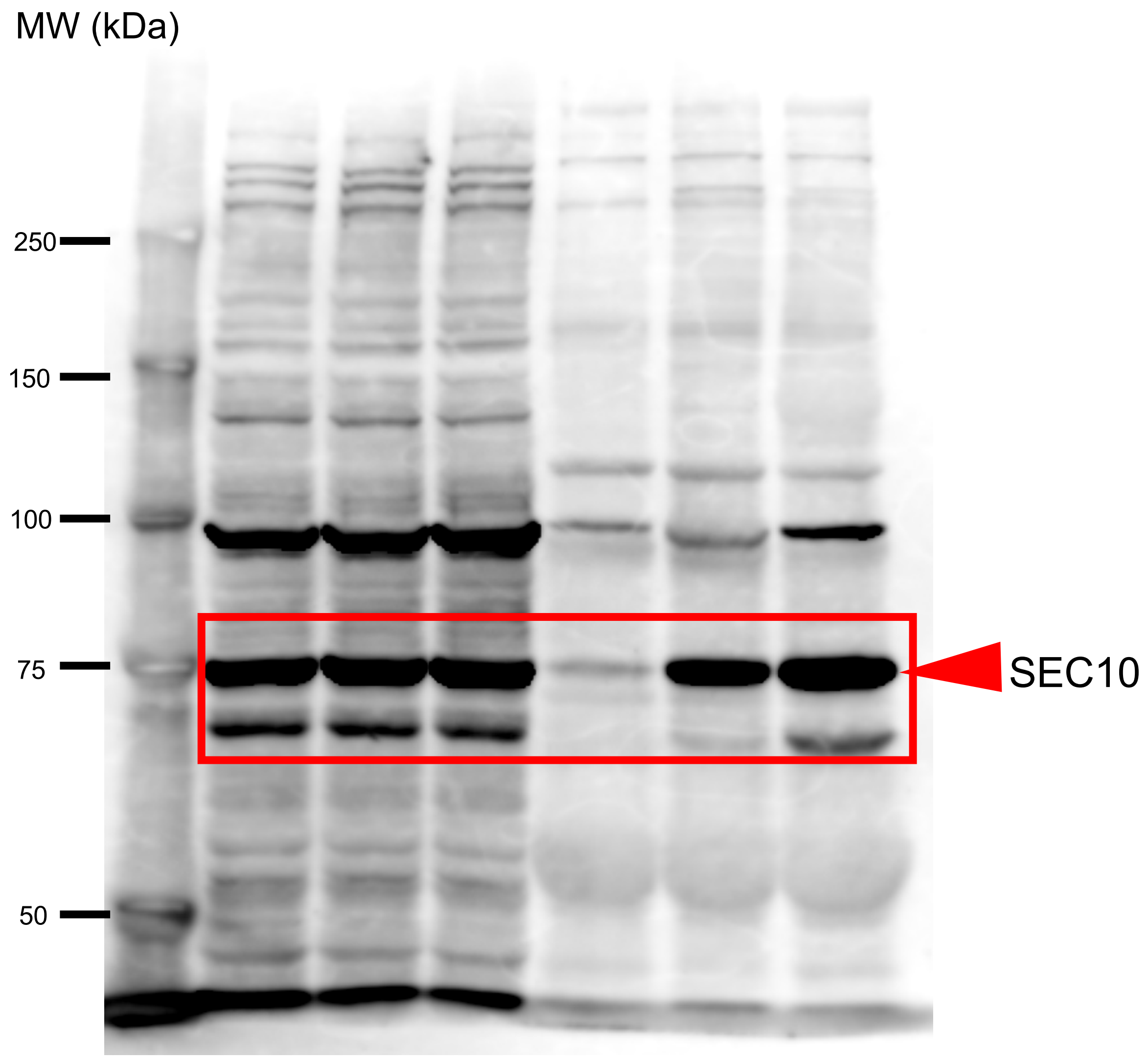


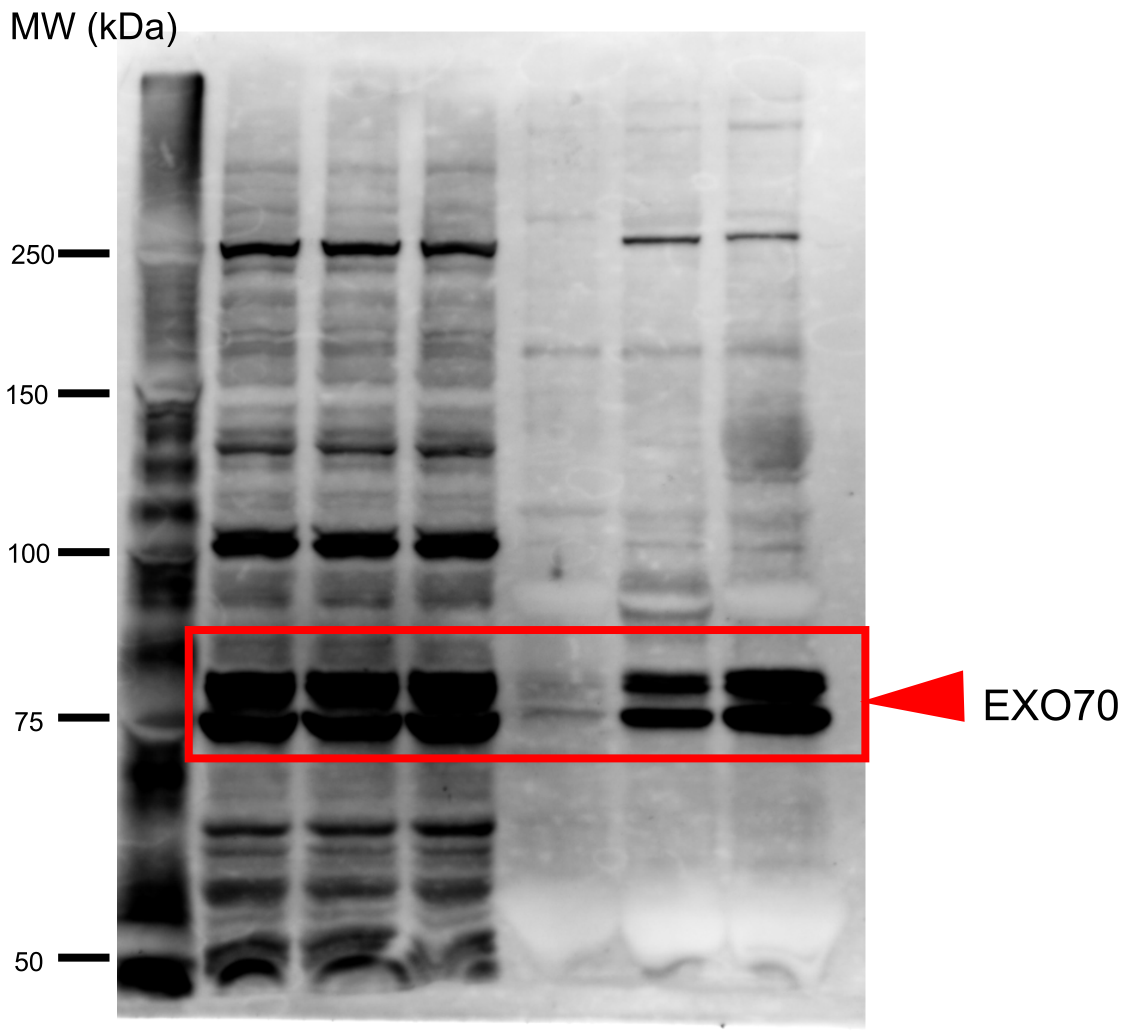

# Source data 2

## Uncropped blot images of Figure 3E

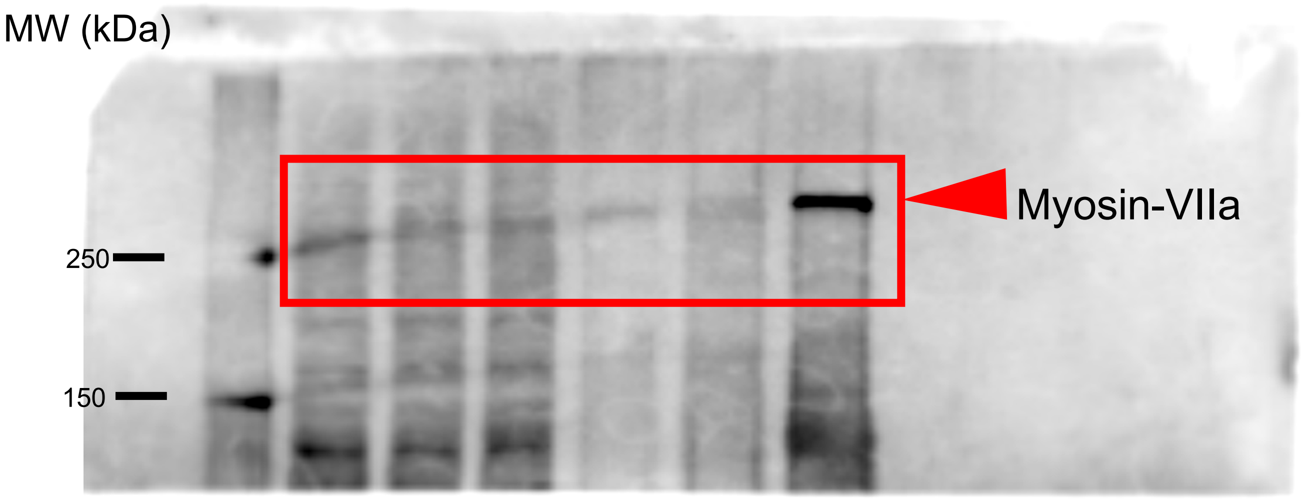


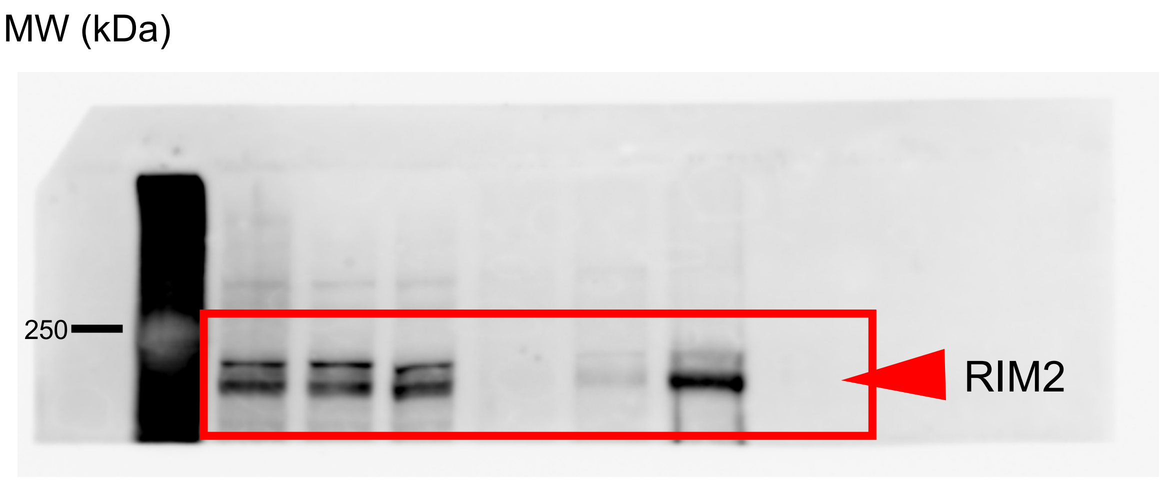

# Source data 2

## Uncropped blot images of Figure 3E

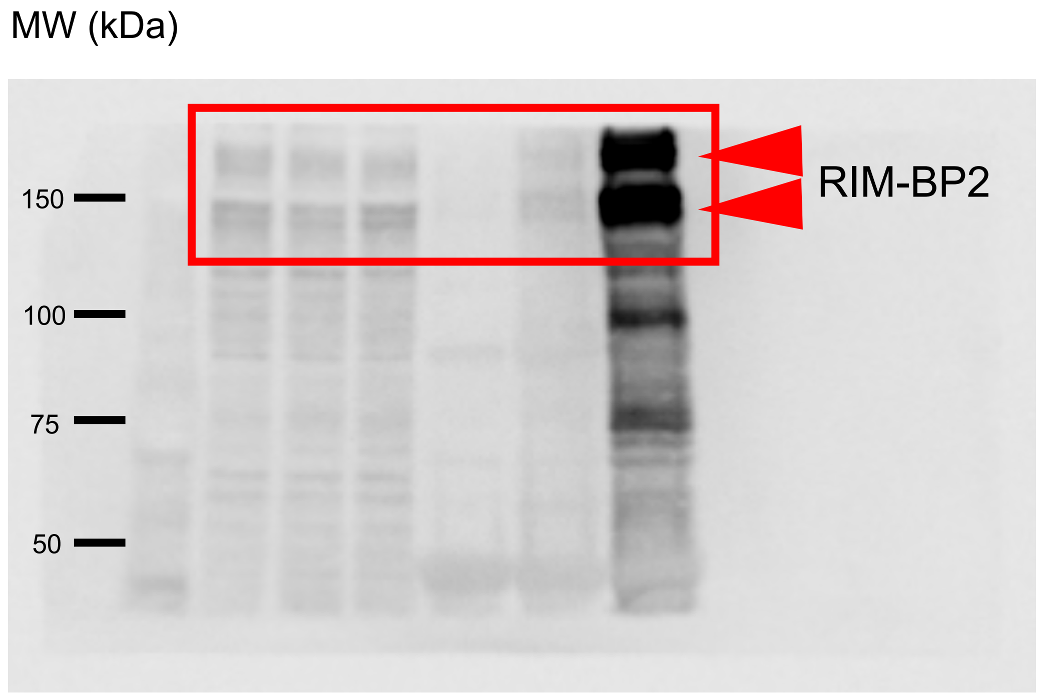


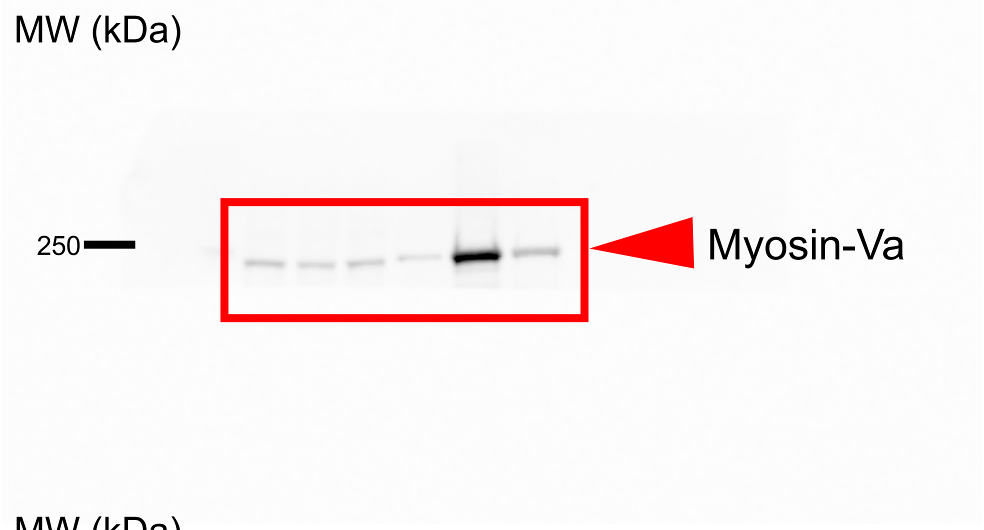


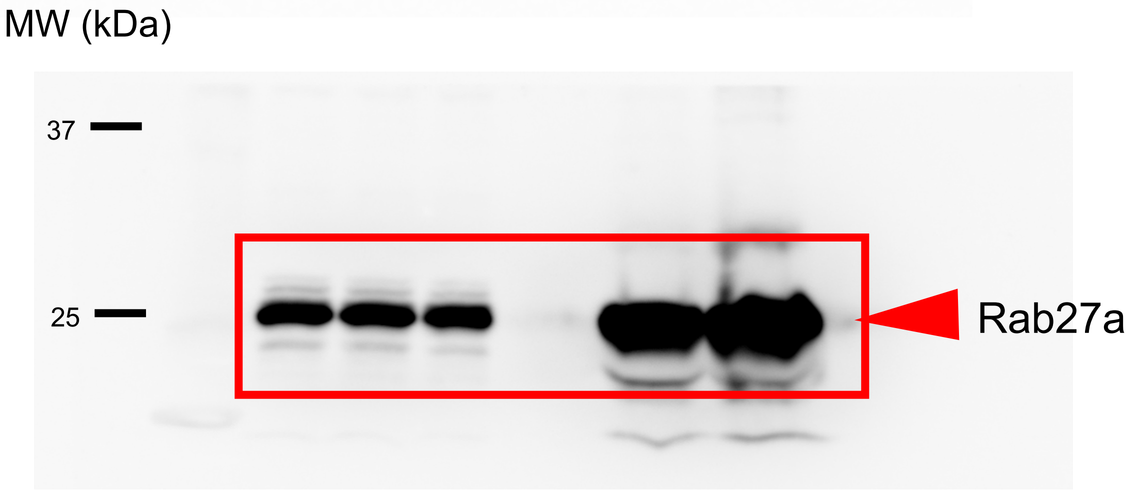

Supplement: Figure 3—source data 1. [file elife-82821-fig3-data1.zip › Figure 3-source data 2/Figure 3-source data 2.pdf]
