# Supplementary material for: Functional hierarchy among different Rab27 effectors involved in secretory granule exocytosis
Source: eLife. 2023 Feb 21;12:e82821. doi: 10.7554/eLife.82821 (PMC9988257; doi:10.7554/eLife.82821)

Source data 4

Uncropped blot images of figure supplement 2B

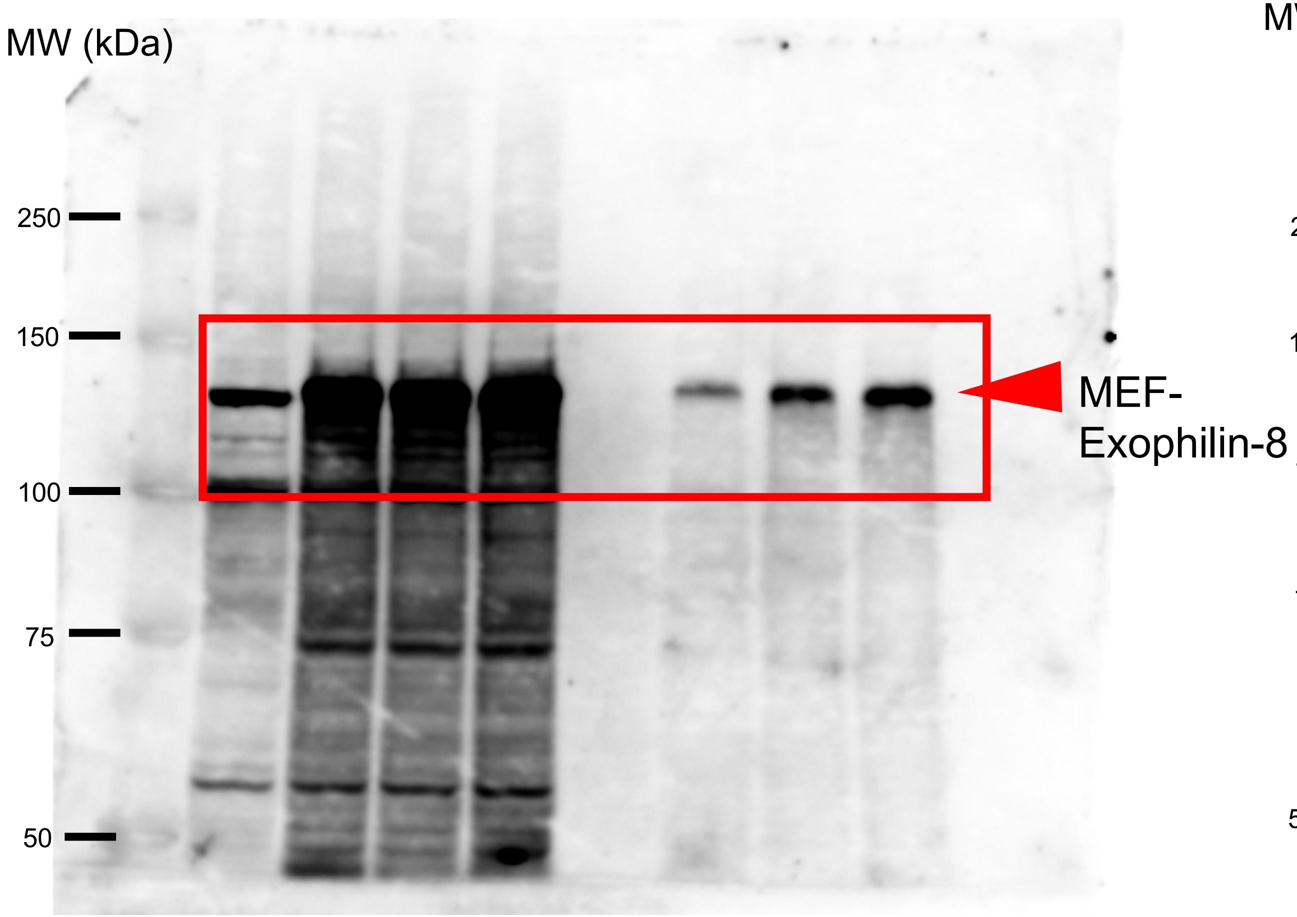

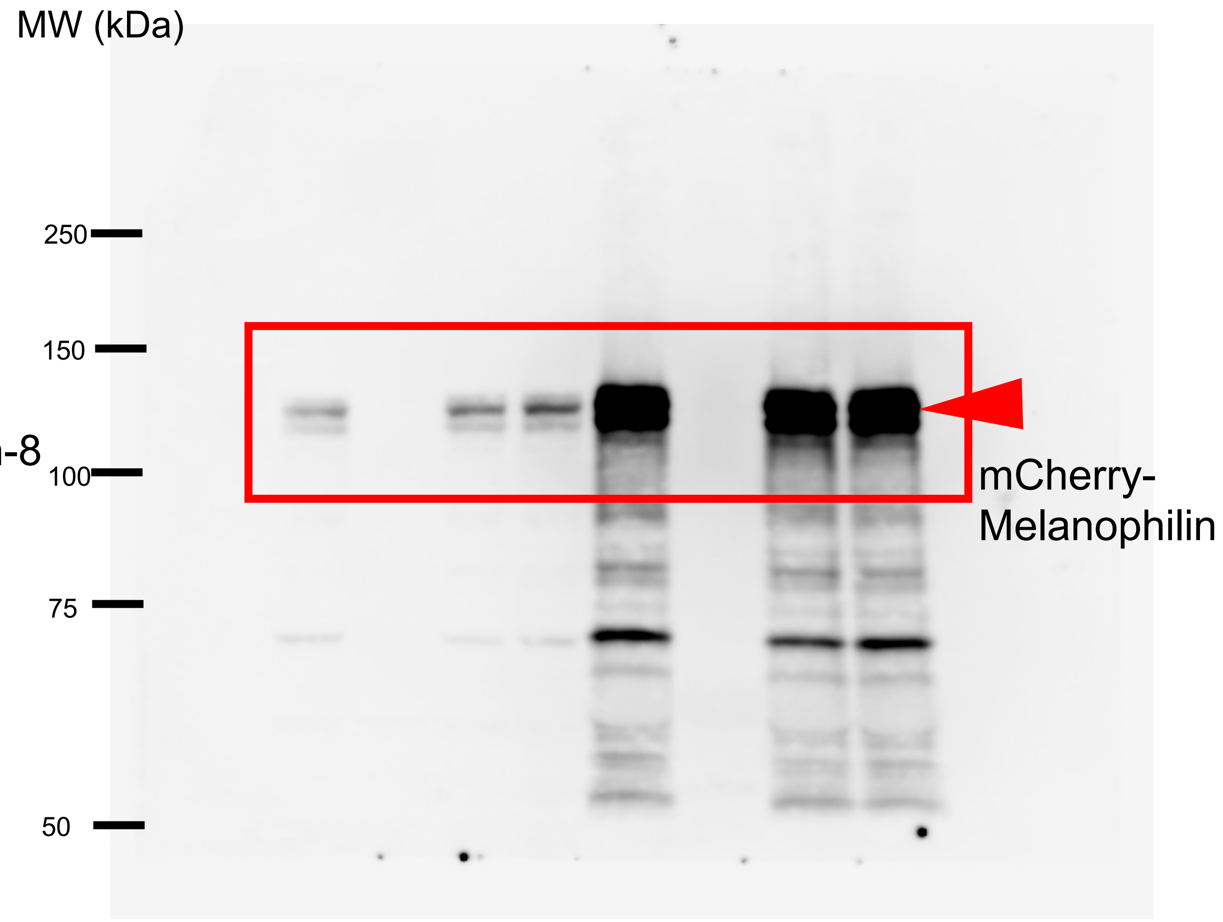

Source data 4

Uncropped blot images of figure supplement 2B

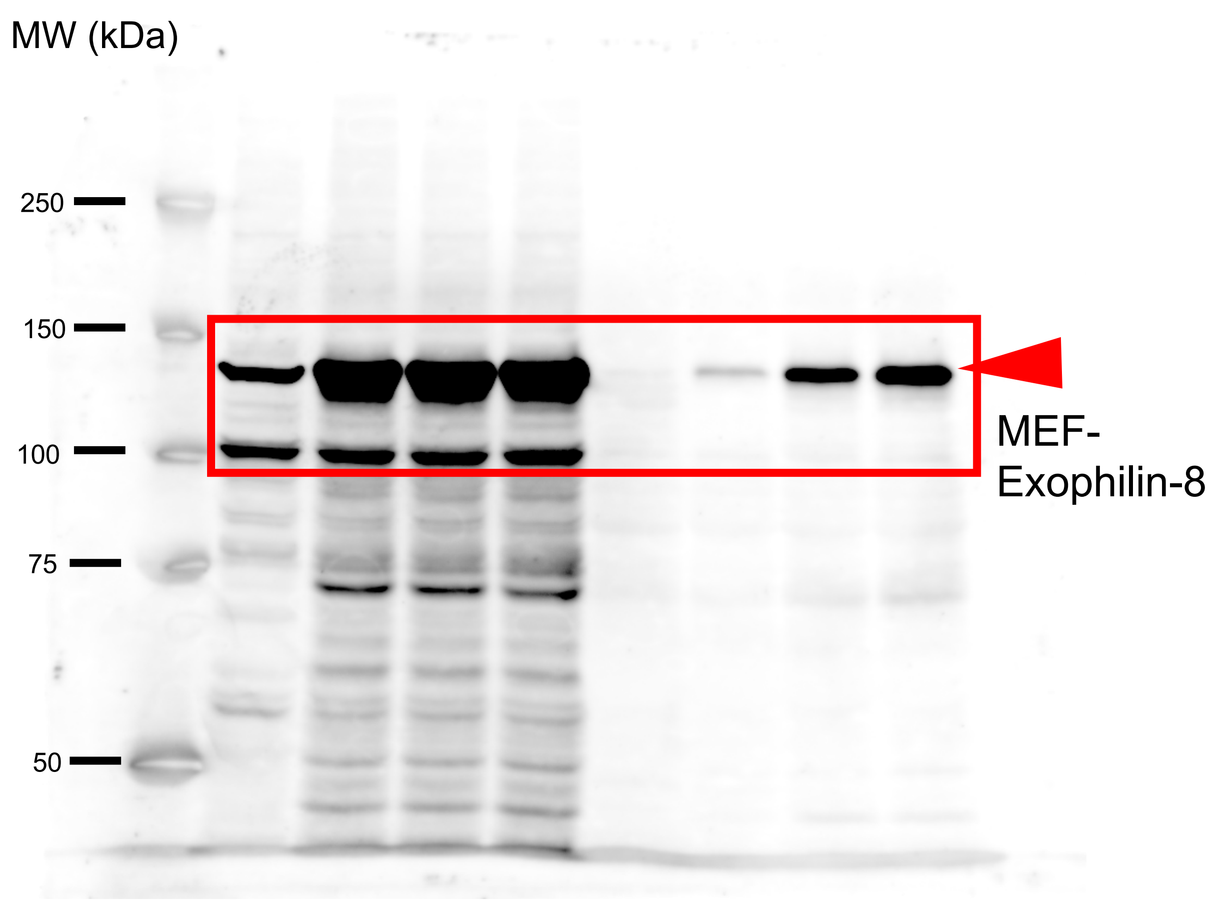

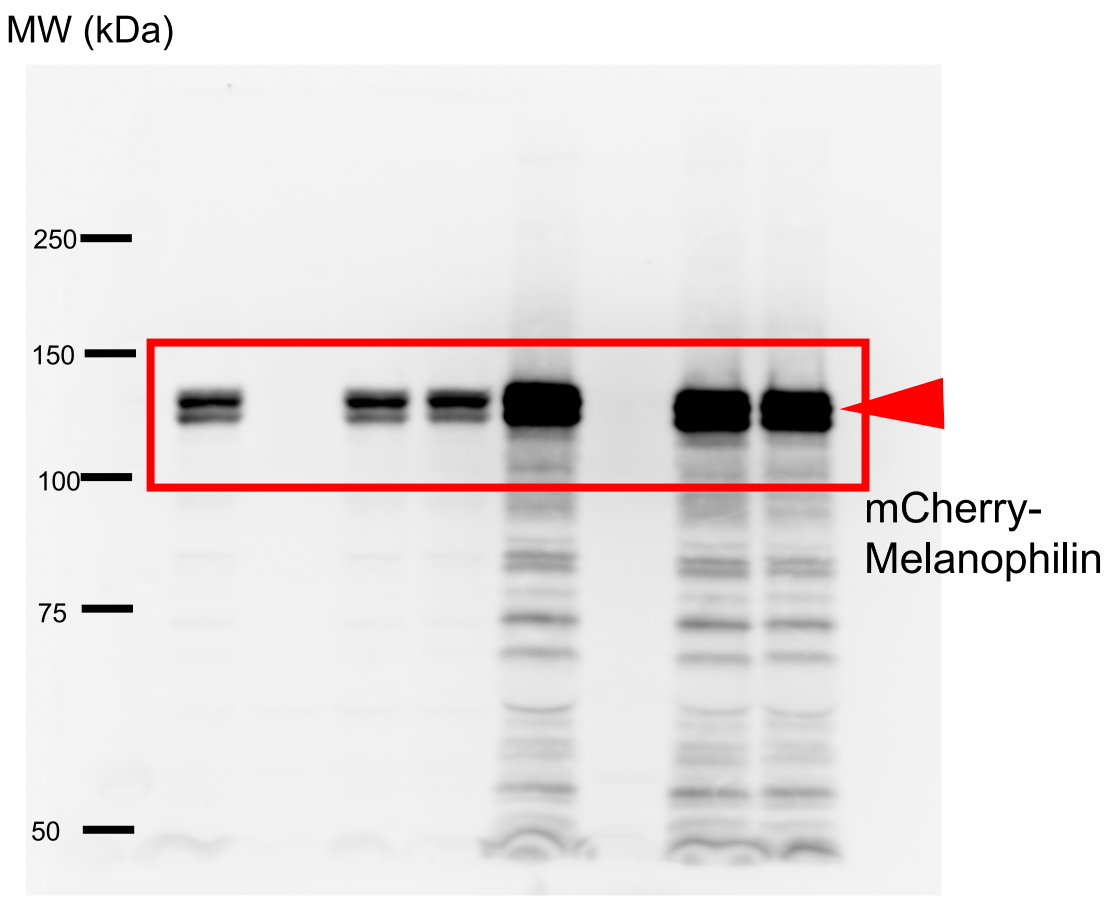

Source data 4

Uncropped blot images of figure supplement 2B

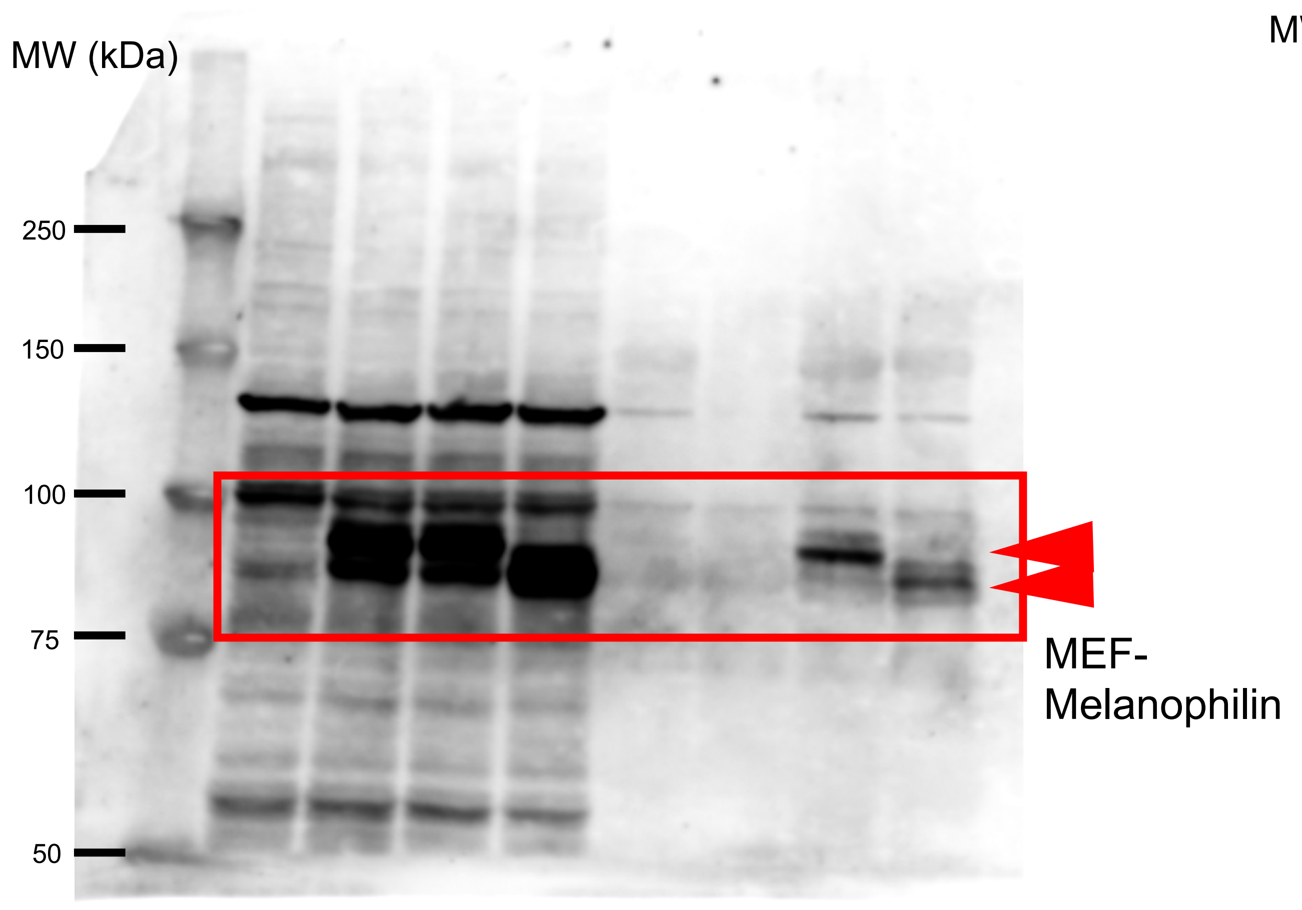

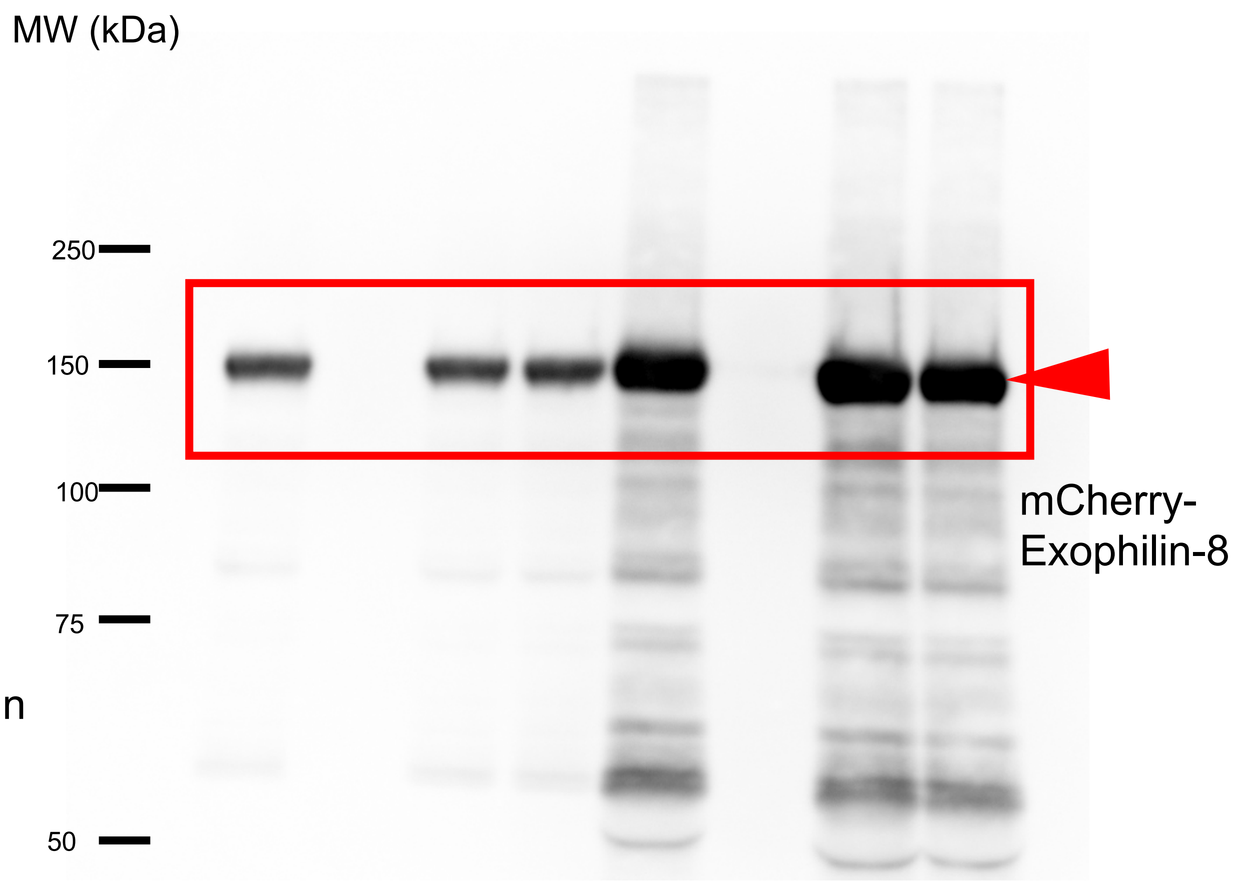

Source data 4

Uncropped blot images of figure supplement 2B

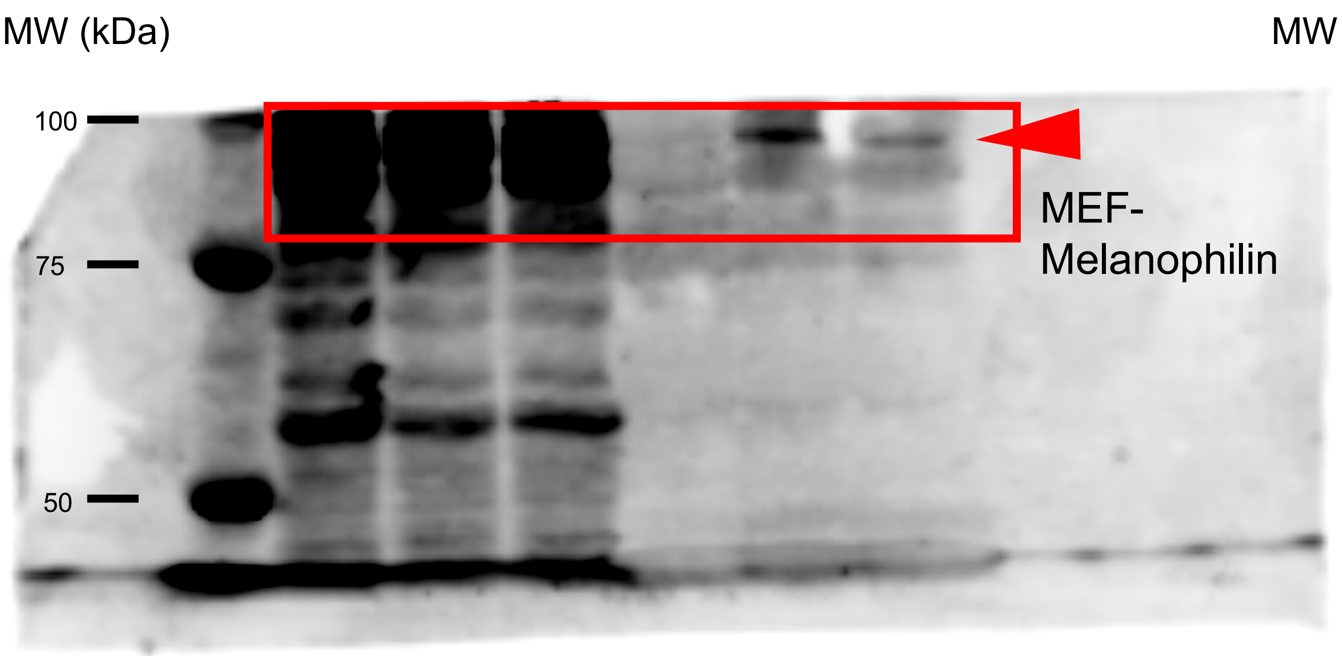

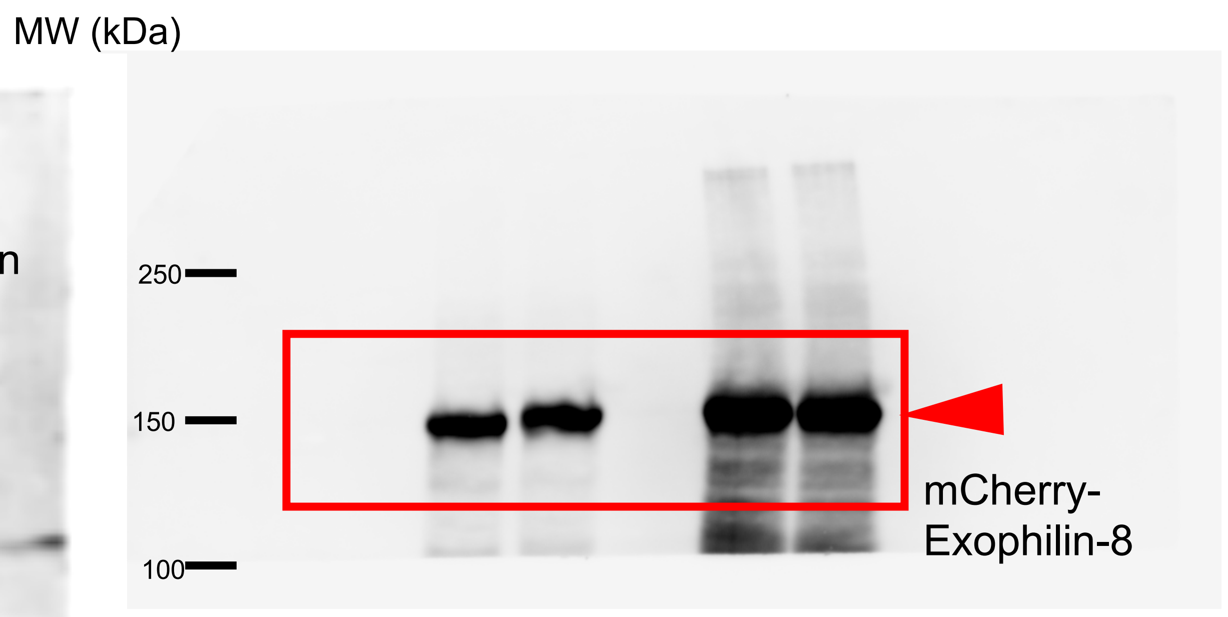

Supplement: Figure 3—figure supplement 2—source data 1. [file elife-82821-fig3-figsupp2-data1.zip › Figure 3-figure supplement 2-source data 4/Figure 3-figure supplement 2-source data 4.pdf]
